# Dual impacts of a glycan shield on the envelope glycoprotein B of HSV-1: evasion from human antibodies *in vivo* and neurovirulence

**DOI:** 10.1128/mbio.00992-23

**Published:** 2023-06-27

**Authors:** Ayano Fukui, Yuhei Maruzuru, Shiho Ohno, Moeka Nobe, Shuji Iwata, Kosuke Takeshima, Naoto Koyanagi, Akihisa Kato, Shinobu Kitazume, Yoshiki Yamaguchi, Yasushi Kawaguchi

**Affiliations:** 1 Division of Molecular Virology, Department of Microbiology and Immunology, The Institute of Medical Science, The University of Tokyo, Tokyo, Japan; 2 Department of Infectious Disease Control, International Research Center for Infectious Diseases, The Institute of Medical Science, The University of Tokyo, Tokyo, Japan; 3 Research Center for Asian Infectious Diseases, The Institute of Medical Science, The University of Tokyo, Tokyo, Japan; 4 Division of Structural Glycobiology, Institute of Molecular Biomembrane and Glycobiology, Tohoku Medical and Pharmaceutical University, Miyagi, Japan; 5 Department of Clinical Laboratory Sciences, School of Health Sciences, Fukushima Medical University, Fukushima, Japan; 6 The University of Tokyo, Pandemic Preparedness, Infection and Advanced Research Center, Tokyo, Japan; The University of North Carolina at Chapel Hill, Chapel Hill, North Carolina, USA; Geisel School of Medicine at Dartmouth, Lebanon, New Hampshire, USA

**Keywords:** herpes simplex virus, immune evasion, glycan shield

## Abstract

Identification of the mechanisms of viral evasion from human antibodies is crucial both for understanding viral pathogenesis and for designing effective vaccines. Here we show in cell cultures that an N-glycan shield on the herpes simplex virus 1 (HSV-1) envelope glycoprotein B (gB) mediated evasion from neutralization and antibody-dependent cellular cytotoxicity due to pooled γ-globulins derived from human blood. We also demonstrated that the presence of human γ-globulins in mice and immunity to HSV-1 induced by viral infection in mice significantly reduced replication in their eyes of a mutant virus lacking the glycosylation site but had little effect on the replication of its repaired virus. These results suggest that an N-glycan shield on a specific site of HSV-1 envelope gB mediated evasion from human antibodies *in vivo* and from HSV-1 immunity induced by viral infection *in vivo*. Notably, we also found that an N-glycan shield on a specific site of HSV-1 gB was significant for HSV-1 neurovirulence and replication in the central nervous system of naïve mice. Thus, we have identified a critical N-glycan shield on HSV-1 gB that has dual impacts, namely evasion from human antibodies *in vivo* and viral neurovirulence.

**IMPORTANCE:**

Herpes simplex virus 1 (HSV-1) establishes lifelong latent and recurrent infections in humans. To produce recurrent infections that contribute to transmission of the virus to new human host(s), the virus must be able to evade the antibodies persisting in latently infected individuals. Here, we show that an N-glycan shield on the specific site of the envelope glycoprotein B (gB) of HSV-1 mediates evasion from pooled γ-globulins derived from human blood both in cell cultures and mice. Notably, the N-glycan shield on the specific site of gB was also significant for HSV-1 neurovirulence in naïve mice. Considering the clinical features of HSV-1 infection, these results suggest that the glycan shield not only facilitates recurrent HSV-1 infections in latently infected humans by evading antibodies but is also important for HSV-1 pathogenesis during the initial infection.

## INTRODUCTION

Herpes simplex viruses (HSV)-1 and HSV-2 cause a variety of human diseases, including encephalitis, keratitis, neonatal disease, and mucocutaneous and skin diseases such as herpes labialis, genital herpes, and herpetic whitlow (1 - 3). A striking feature of these viruses is that they establish lifelong infections in humans, where, after the initial infection, they become latent and frequently reactivate to cause lesions (1 - 3). To accomplish these cycles, the viruses have evolved highly complex and sophisticated strategies to evade host immune mechanisms. Notably, these viral strategies have probably impeded the development of effective vaccines for HSV-1 and HSV-2 infections. Several decades of vaccine development have not produced a successful vaccine (4, 5).

To clarify the significance of the mechanisms of immune evasion by viruses that cause diseases in humans, the mechanisms should be investigated not only *in vitro* but also *in vivo*; and research using available human samples should provide valuable information on the effective viral mechanisms in humans. However, in previous studies, human samples have generally been analyzed *in vitro*, and information from the *in vivo* evaluations of human samples on viral evasion from the immune system has been limited. To fill the gaps in our understanding of the effective mechanisms of viral immune evasion in humans, *in vivo* investigations of the mechanisms of immune evasion that use human samples are of crucial importance.

Although no effective vaccines for HSV-1 and HSV-2 have been developed thus far, previous clinical trials for HSV vaccines have provided important clues indicating that not only T-cell responses, but also antibody responses were important for controlling HSV infections in humans (4, 5). Thus, a clinical trial of a subunit vaccine employing HSV-2 envelope glycoprotein D (gD) showed partial but consistent efficacy against the development of HSV-1 genital disease but did not offer significant protection against HSV-2 genital disease (6). Notably, antibody responses to HSV-2 gD correlated with protection against HSV-1 but not HSV-2 infections, whereas CD4^+^ T-cell responses did not correlate with protection against either HSV-1 or HSV-2 infections (7). In addition, a substudy of this trial, which used sera from a fraction of the vaccinated subjects, showed that neutralizing antibody titers against HSV-1 were significantly higher than the titers against HSV-2 (8). These findings were in agreement with those from another clinical study in humans that showed that the absence of HSV antibodies was associated with severe HSV infections in humans (9). It is of interest that the response to the HSV-2 gD subunit vaccine was restricted to the generation of gD neutralizing antibodies and that antibody-dependent cellular cytotoxicity (ADCC) did not develop (10).

The findings described in the previous paragraph suggesting that antibodies are important for the control of HSV-1 infections led us to attempt to identify hitherto unknown mechanisms of HSV-1 evasion from human antibodies. In this study, we focused on the glycosylation of a major envelope glycoprotein of HSV-1, gB. Glycosylation of a viral envelope glycoprotein sometimes acts as a glycan shield(s) that evades antibodies (11). This mechanism of antibody evasion has been documented for influenza virus, human immunodeficiency virus, Nipah virus, hepatitis C virus, Ebola virus, hepatitis B virus, lymphocytic choriomeningitis virus, and porcine reproductive and respiratory syndrome virus (12 - 20).

HSV-1 gB, which is a class III fusion glycoprotein, is a major target of antibody-mediated immunity (21). It plays an essential role in the entry of the virus into a host cell, together with other HSV-1 envelope glycoproteins, including gD and a complex of gH and gL (gH/gL) (22). Herein, we investigated the effects of a series of N-linked glycans (N-glycans) on HSV-1 gB in the context of viral infection and identified an N-glycan that contributed to evasion from human antibodies not only *in vitro* but also *in vivo*. Notably, the N-glycan on gB was also significant for HSV-1 replication in the central nervous system (CNS) of naïve mice as well as neurovirulence, although it had no effect on viral replication in cell cultures.

## RESULTS

### Generation of recombinant viruses harboring a mutation in each of the potential N-glycosylation sites on HSV-1 gB by an improved genetic manipulation system for HSV-1

**FIG 1:**
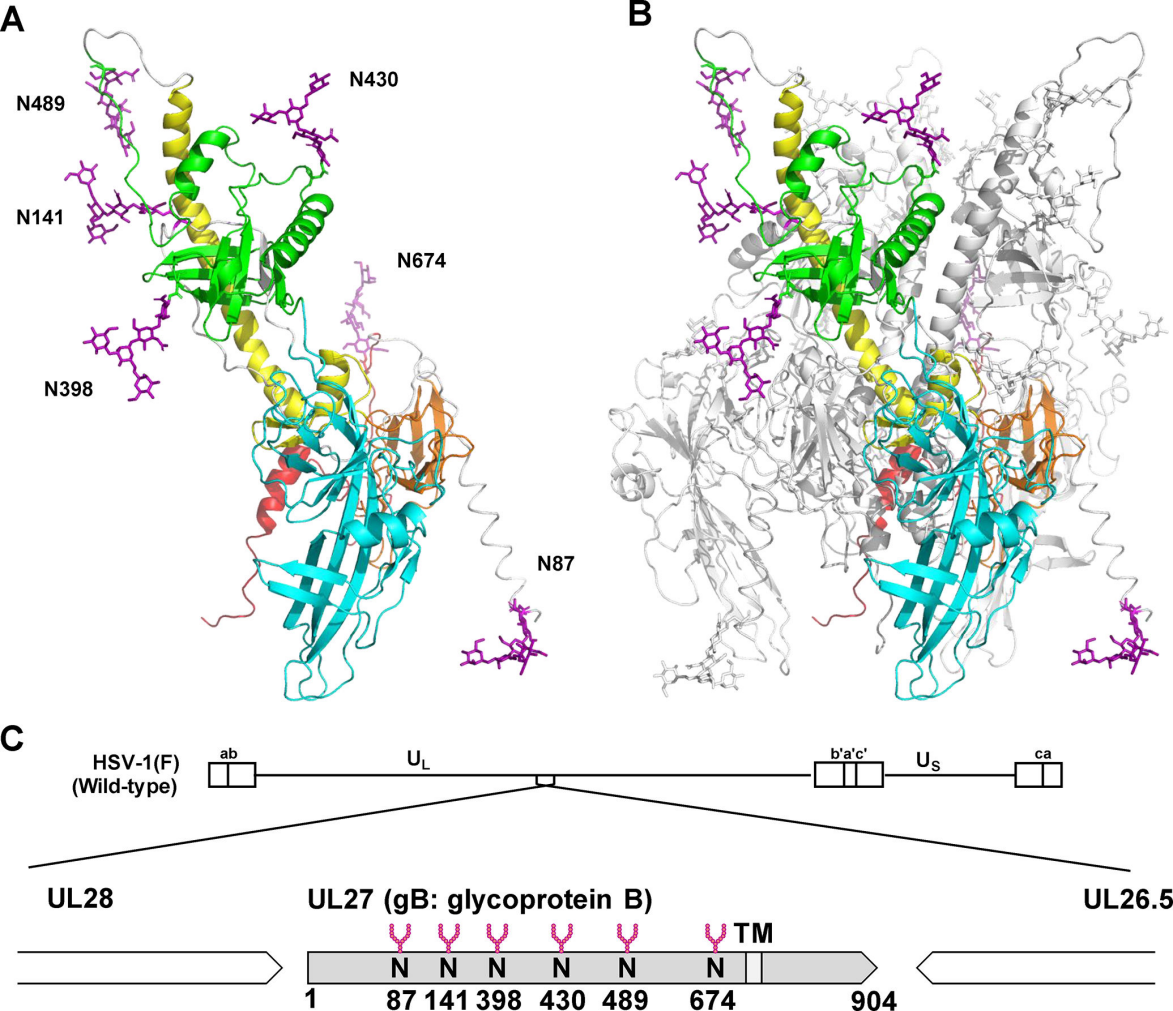
A three-dimensional (3D) structural model of fully N-glycosylated gB in the prefusion state. Image A shows the ribbon diagram of the protomer, and image B shows the trimer of the crystal structure of gB in the prefusion state (Protein Data Bank accession no. 6Z9M) (23). The Glycan Reader and Modeler were used to modify potential N-glycosylation sites (Asn-87, Asn-141, Asn-398, Asn-430, Asn-489, and Asn-674) of prefusion gB with the Man_3_GlcNAc_2_ glycan using Glycan Modeler. Missing coordinates of Asn-87 and Asn-489 in the 3D structure were estimated. The ribbon diagrams of the gB models show the functional domains in colors as follows: domain I (light blue), domain II (green), domain III (yellow), domain IV (orange), and domain V (red). Residue background coloring is used for the main polypeptide chains, and potential N-glycans are shown in magenta as stick models. In Panel B, protomer A is the same as in Panel A. Protomer B and C are shown in white. Image C shows the location of potential N-linked glycans on gB along the genome of wild-type HSV-1(F). N, N-glycosylation sites (Asn-87, Asn-141, Asn-398, Asn-430, Asn-489, and Asn-674); TM, transmembrane domain.

HSV-1 gB has six potential N-glycosylation sites at the following positions: Asn-87, Asn-141, Asn-398, Asn-430, Asn-489, and Asn-674 (Fig. 1). To investigate the significance of N-glycosylation on HSV-1 gB in the context of viral infection, we used an improved HSV-1 genetic manipulation system to construct a series of recombinant viruses and their repaired viruses (Fig. S1). The recombinant viruses encoded mutant gBs (gB-N87Q, gB-N141Q, gB-N398Q, gB-N430Q, gB-N489Q, and gB-N674Q), in which each of the potential N-glycosylation sites was substituted with glutamine. In addition, we generated a pair of control viruses, a recombinant virus, in which Asn-888 in the cytoplasmic domain of gB was substituted with glutamine (gB-N888Q), and its repaired virus (gB-N888Q-repair) (Fig. S1).

The two-step Red-mediated recombination system consists of the first recombination for the insertion of a PCR-amplified selectable marker and the second recombination for the excision of the inserted marker by a cleavage step that uses a rare-cutting endonuclease I-SceI. This system is widely used for markerless modifications of large DNA molecules such as herpesvirus genomes that are cloned into a bacterial artificial chromosome (BAC) in *Escherichia coli* (24).

However, the second recombination step was not very efficient in our hands. Therefore replica plating, which is time-consuming and laborious, was needed to identify any *E. coli* recombinant harboring a BAC clone with a desired mutation. We used an improved system that was developed for this study that employed a negative selection marker, the *E. coli* phenylalanyl-tRNA synthetase (ePheS*), which encodes a mutant of the α-subunit of *E. coli* phenylalanyl-tRNA synthetase (ePheS) (25) in the presence of 4-chloro-phenylalanine (4-CP), in addition to cleavage by I-SceI for the second recombination step (Fig. S2A). The improved system considerably increased the efficiency of the second recombination step from 17.4% to 87%, 17.4% to 94.7%, and 8.7% to 87.0% in the substitution of single amino acids, and in the deletion and insertion of a foreign gene, respectively, compared with the original system (Table S1A). Recombinant viruses carrying an alanine substitution of Thr-190 in the HSV-1 protein UL51 (Fig. S3A), which were generated in the original and improved systems, exhibited identical growth properties (Fig. S3B) in Vero cells and produced identical neurovirulence in mice following intracranial inoculation (Fig. S3C), suggesting that, compared with the original system, the additional negative selection in the improved system did not affect the genomic integrity of HSV-1 other than the desired mutations.

### Effects of mutations at each of the potential gB N-glycosylation sites on electrophoretic mobility in the presence or absence of peptide-N-glycosidase F (PNGase)

**FIG 2:**
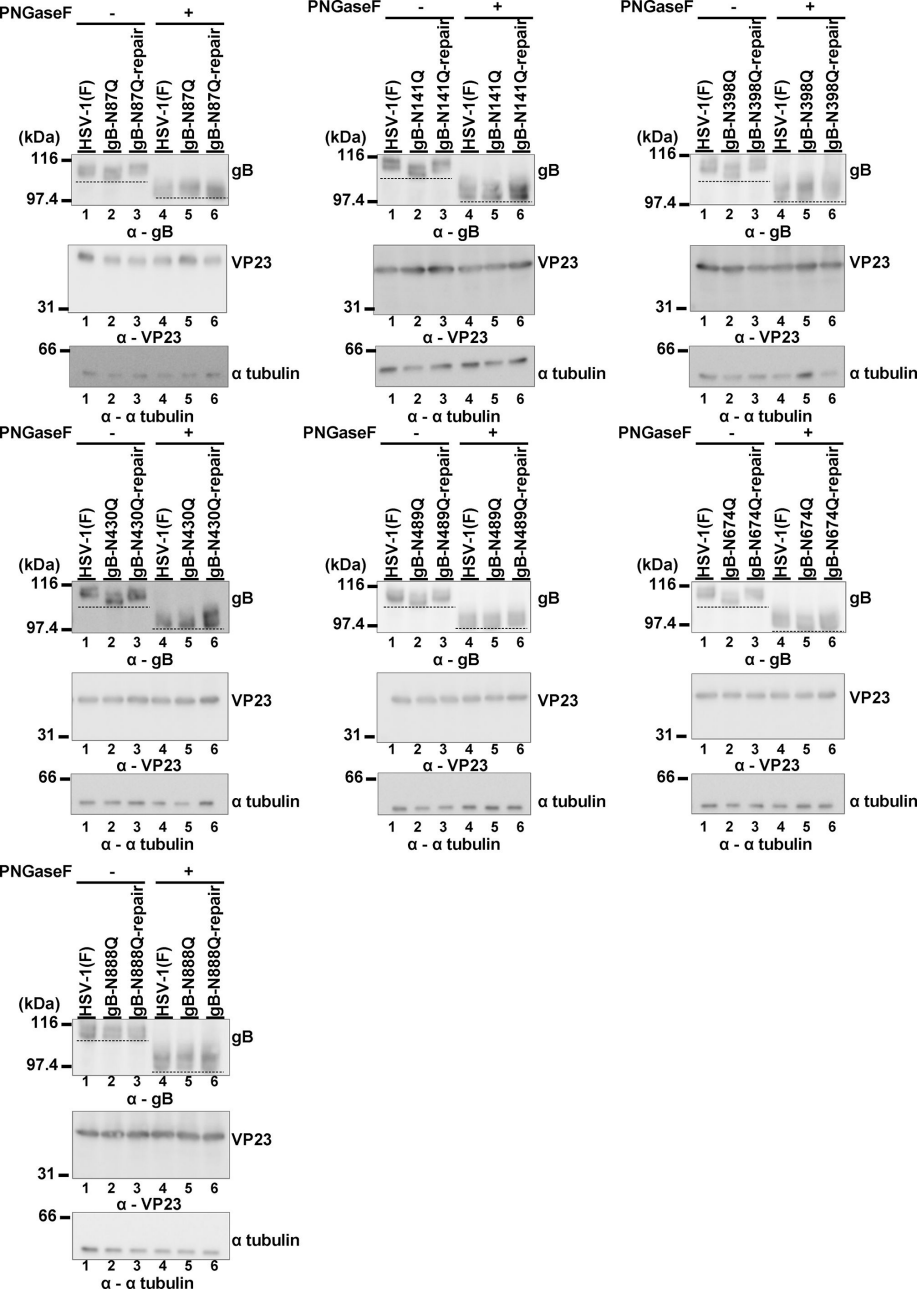
Effect of mutation at each of the potential gB N-glycosylation sites on electrophoretic mobility in the presence or absence of PNGase. Vero cells infected for 21 h with wild-type HSV-1(F), each of the gB mutant viruses, or each of their repaired viruses at an MOI of 5 were lysed, treated with or without PNGase, and analyzed by immunoblotting with antibodies to gB, VP23, or α-tubulin. Data are representative of three independent experiments. Dashed lines indicate the bottom of bands harboring gB with each of the indicated NQ mutations. α, anti.

Vero cells infected with wild-type HSV-1(F), each of the gB mutant viruses, or each of their repaired viruses were lysed, treated with or without PNGase, and analyzed by immunoblotting. As shown in Fig. 2, all of the gB mutants, except the gB-N888Q mutant, migrated faster than wild-type gB in denaturing gels. In contrast, the gB-N888Q mutant and gB from cells infected with each of the repaired viruses migrated as slowly as the wild-type gB in denaturing gels (Fig. 2). After treatment of the infected cell lysates with PNGase, all of the gB mutants migrated in denaturing gels as slowly as the wild-type gB (Fig. 2). All of the gB mutants were detected by immunoblotting at levels similar to the level of wild-type gB (Fig. 2). These results indicate that gB was N-glycosylated at each of the six potential N-linked glycosylation sites without affecting its accumulation in HSV-1-infected cells.

### Effects of mutations at each of the gB N-glycosylation sites on the replication of HSV-1 in cell cultures

To investigate the effect of gB N-glycosylation on HSV-1 replication in cell cultures, Vero cells were infected with wild-type HSV-1(F), each of the gB mutant viruses, or each of their repaired viruses at a multiplicity of infection (MOI) of 5 or 0.01; and virus titers were assayed at 24 or 48 h postinfection. As shown in Fig. S4, progeny virus yields in cells infected with each of the gB mutant viruses were similar to those in cells infected with wild-type HSV-1(F) or each of their repaired viruses. These results suggest that N-glycosylation on gB did not affect HSV-1 replication in cell cultures.

### Effects of mutations at each of the gB N-glycosylation sites on viral susceptibility to neutralization by human antibodies

An estimated 67% of the global human population is infected with HSV-1 (26). Therefore, we decided to use pooled γ-globulins from human blood that contain amounts of antibodies to HSV-1 sufficient for our experiments (27). Indeed, we showed that gB antibodies in pooled human γ-globulins at a concentration of 1.3 mg/mL could still be detected at a dilution of 1:1024, whereas gD antibodies could be detected at a dilution of 1:16384 (Fig. S5). To investigate the effect of gB N-glycosylation on viral susceptibility to neutralization by human antibodies, the sensitivity to neutralization of wild-type HSV-1(F) by pooled human γ-globulins was compared to each of the recombinant gB mutant viruses. Among the gB mutant viruses tested, gB-N141Q was only the gB mutant virus that was significantly more susceptible to neutralization by pooled human γ-globulins at a concentration (dilution) of 0.041 mg/mL (1:32) compared with wild-type HSV-1(F) (Fig. S6). Therefore, we focused on N-glycosylation at gB Asn-141 and further characterized gB-N141Q in detail.

**FIG 3:**
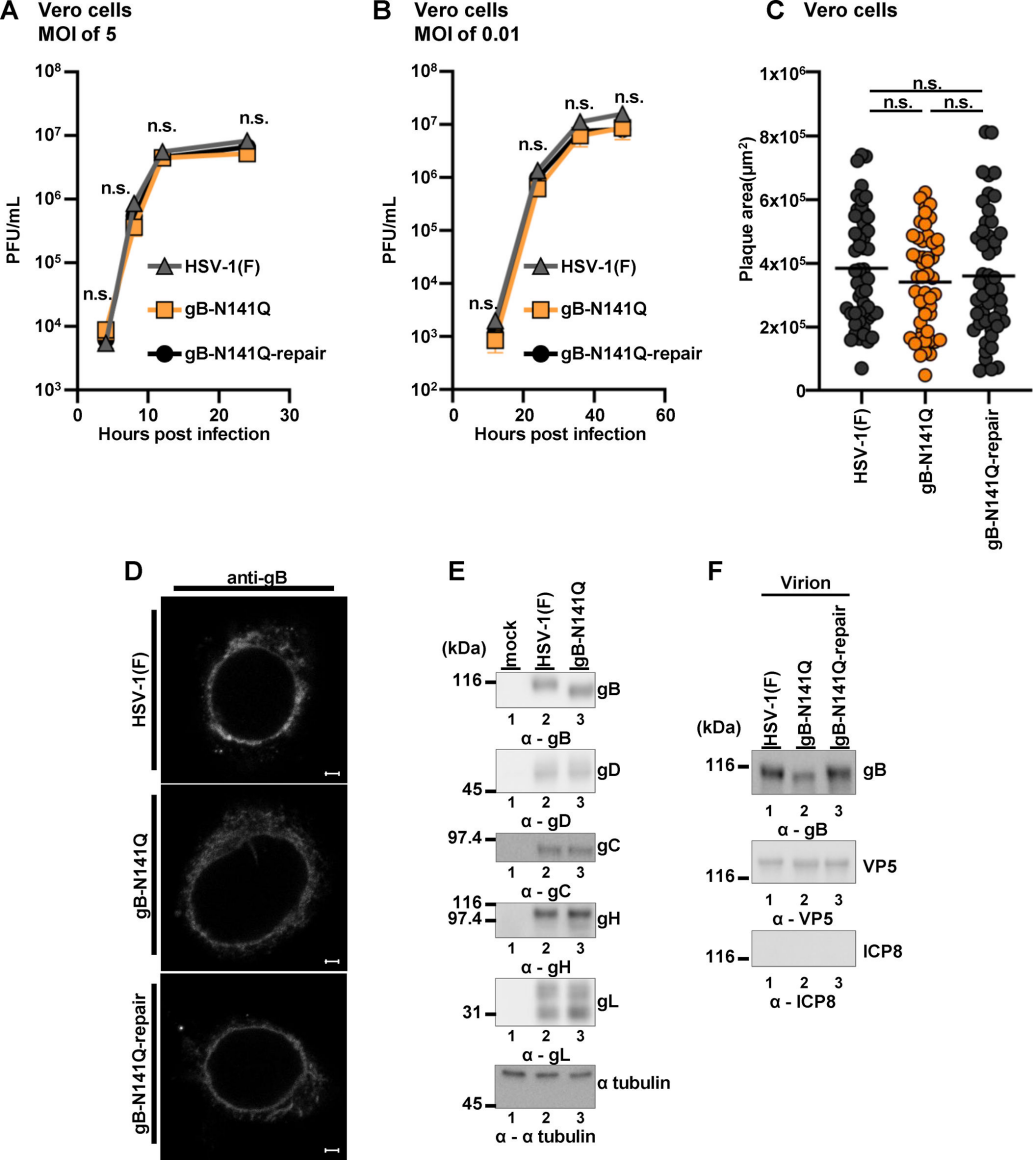
Characterization of the recombinant viruses YK683 (gB-N141Q) and YK684 (gB-N141Q-repair). (A and B) Vero cells were infected with wild-type HSV-1(F), gB-N141Q, or gB-N141Q-repair viruses at an MOI of 5 (A) or 0.01 (B). Total virus from the cell culture supernatants and infected cells was harvested at the indicated times and assayed on Vero cells. Each value is the mean ± standard error of the results of three independent experiments. (C) Vero cells were infected with 200 PFU of wild-type HSV-1(F), gB-N141Q, or gB-N141Q-repair, as described in Materials and Methods. The areas of 50 individual plaques produced by each of the indicated viruses were measured 2 days postinfection. The horizontal bars indicate the mean area of each group of plaques. (D) Vero cells infected with wild-type HSV-1(F), gB-N141Q, or gB-N141Q-repair at an MOI of 5 and incubated for 18 h were fixed, permeabilized, stained with antibody to gB, and examined by confocal microscopy. Scale bars = 2 μm. (E) Vero cells infected with wild-type HSV-1(F) or gB-N141Q at an MOI of 5 and incubated for 21 h were lysed, and analyzed by immunoblotting with antibodies to gB, gD, gC, gH, gL, or α-tubulin. (F) Vero cells were infected with wild-type HSV-1(F), gB-N141Q, or gB-N141Q-repair at an MOI of 0.01 and incubated for 2 days. Purified virions from the infected cells were analyzed by immunoblotting. The data are representative of three independent experiments (D, E, and F). Statistical analysis was performed by one-way ANOVA followed by the Tukey test (A, B, and C). n.s., not significant; α, anti, ANOVA, analysis of variance.

As shown in Fig. 3A and B, the growth kinetics of gB-N141Q in Vero cells at MOIs of 5 and 0.01 were almost identical to those of wild-type HSV-1(F) or gB-N141Q-repair. Similarly, gB-N141Q produced plaques in Vero cells of similar size to the sizes of plaques produced by wild-type HSV-1(F) or gB-N141Q-repair (Fig. 3C). Confocal microscopy showed that the subcellular locations of gB in cells infected with either gB-N141Q, wild-type HSV-1(F), or gB-N141Q-repair were also similar (Fig. 3D). Furthermore, Vero cells infected with gB-N141Q accumulated other envelope glycoproteins, including gD, gC, gH, and gL, at levels similar to the levels in cells infected with wild-type HSV-1(F) (Fig. 3E). We observed that the amounts of gB-N141Q in the mutant virions were slightly lower than the amounts of wild-type gB in the wild-type and gB-N141Q-repair virions (Fig. 3F).

**FIG 4:**
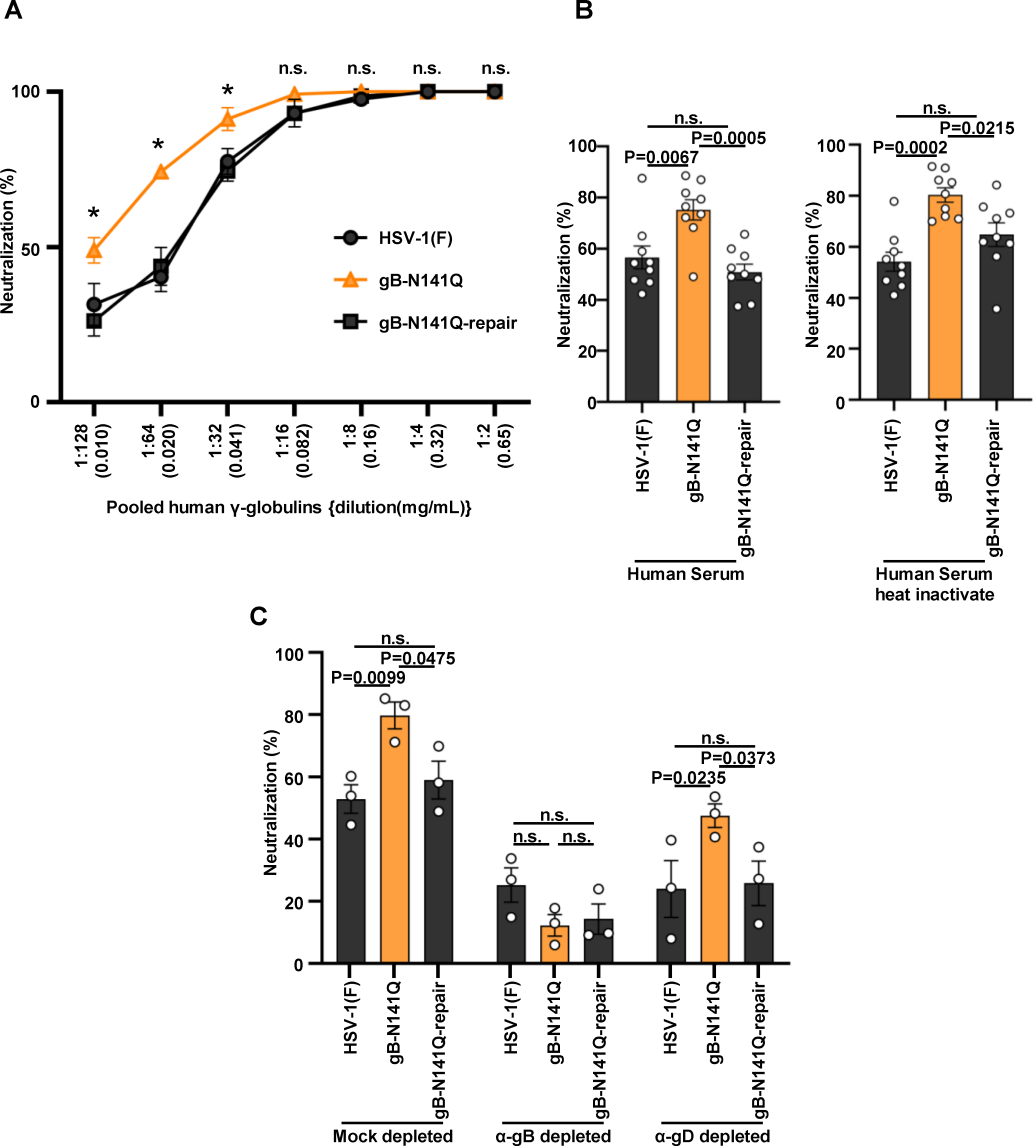
Effect of N-glycan at gB Asn-141 on viral susceptibility to neutralization by pooled human γ-globulins. (A) 100 PFU of wild-type HSV-1(F), gB-N141Q, or gB-N141Q-repair was incubated with serially diluted human γ-globulins at 37°C for 1 h and then inoculated onto Vero cell monolayers for plaque assays. The percentage of neutralization was calculated from the number of plaques formed by each of the viruses that were incubated with or without human γ-globulins, as follows: 100 × [1 − (number of plaques after incubation with human γ-globulins)/(number of plaques after incubation without human γ-globulins)]. (B) 100 PFU of wild-type HSV-1(F), gB-N141Q, or gB-N141Q-repair was incubated with 1:500 diluted human serum or heat-inactivated human serum at 37℃ for 1 h and then inoculated onto Vero cell monolayers as described in A. (C) Human γ-globulins (0.082 mg/mL) were mock depleted or depleted with gB-SE (α-gB depleted) or gD-SE (α-gD depleted) as shown in Fig. S7. Wild-type HSV-1(F), gB-N141Q, or gB-N141Q-repair was incubated with each of the depleted human γ-globulins and then inoculated onto Vero cell monolayers as described in A. Each value represents the mean ± standard error of the results of three (A and C) or nine (B) independent experiments. Statistical analysis was performed by two-way analysis of variance (ANOVA) followed by the Tukey test (A and C) or by one-way ANOVA followed by the Tukey test (B). *, *P* < 0.05 indicates statistically significant differences between gB-N141Q and wild-type HSV-1(F) or gB N141Q-repair; n.s., not significant; α, anti.

When incubated in dilutions of pooled human γ-globulin ranging from 0.010 (1:128) to 0.041 mg/mL (1:32), gB-N141Q proved to be significantly more susceptible to neutralization by human γ-globulins than wild-type HSV-1(F) (Fig. 4A). Wild-type susceptibility was restored in the gB-N141Q-repair virus (Fig. 4A). Similarly, gB-N141Q was significantly more susceptible to neutralization by heat-inactivated pooled human sera than wild-type HSV-1(F) and gB-N141Q-repair (Fig. 4B). Heat inactivation of the pooled human sera had little effect on the degree of increased susceptibility to neutralization by the N141Q mutation in gB (Fig. 4B). Antibodies to gB or gD in the pooled human γ-globulins [0.082 mg/mL (1:16)] were then depleted by treatment of the pooled human γ-globulins with purified gB or gD fused with Strep-tag at the C-terminus (gB-SE or gD-SE, respectively) (Fig. S7A and B). The anti-gB antibody-depleted human γ-globulins could not detect gB ectopically expressed by HEK293FT cells (Fig. S7C and D). As shown in Fig. 4C, the susceptibility of gB-N141Q to neutralization by anti-gB antibody-depleted human γ-globulins (~0.4 mg/mL) was comparable to that of wild-type HSV-1(F) and gB-N141Q-repair. In contrast, gB-N141Q was significantly more susceptible to neutralization by mock-depleted or anti-gD antibody-depleted human γ-globulins than wild-type HSV-1(F) and gB-N141Q-repair (Fig. 4C), as seen in Fig. 4A, showing its susceptibility to human γ-globulins without antibody depletion. These results suggest that the N-glycan on gB Asn-141 was required for efficient HSV-1 evasion from complement-independent neutralization by human antibodies that targeted gB in cell cultures.

### Effects of the N-glycan on gB Asn-141 on human ADCC

**FIG 5:**
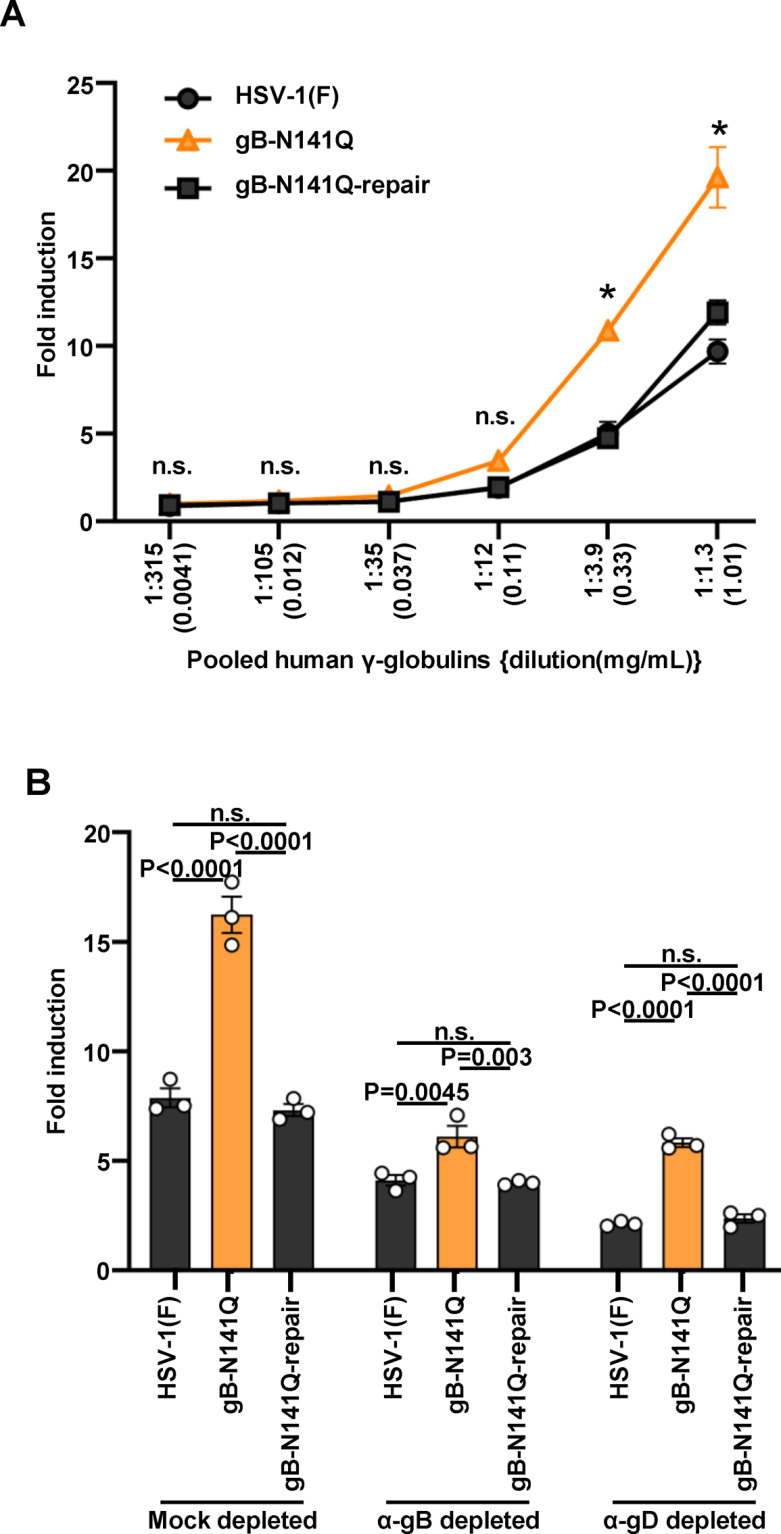
Effects of the N-glycan at Asn-141 on gB on the extent of ADCC induced by human γ-globulins. (A) Vero cells were infected with wild-type HSV-1(F), gB-N141Q, or gB-N141Q-repair at an MOI of 1 for 24 h and co-cultured with ADCC effector cells in the presence or absence of serially diluted human γ-globulins for 6 h. A luciferase assay was then performed. (B) Human γ-globulins (3.04 mg/mL) were mock depleted or depleted with gB-SE (α-gB depleted) or gD-SE (α-gD depleted) as depicted in Fig. S7 and used as described in 4A. Values are fold induction relative to controls without antibody. Each value is the mean ± standard error of the results of three biologically independent samples. The statistical analysis was performed by two-way ANOVA followed by the Tukey test. *, *P* < 0.05 indicates statistically significant differences between gB-N141Q and wild-type HSV-1(F) or gB-N141Q-repair; n.s., not significant; α, anti; ANOVA, analysis of variance.

It has been reported that gB on the surface of infected cells mediates ADCC (28); therefore, we examined the effect of the N-glycan on gB Asn-141 on ADCC induced by human γ-globulins. Vero cells infected with wild-type HSV-1(F), gB-N141Q, or gB-N141Q-repair at an MOI of 1 for 24 h were subjected to an activating Fcγ receptor (R) IIIA ADCC assay in the presence or absence of human γ-globulins. As shown in Fig. 5A, human γ-globulins at concentrations (dilutions) of 0.33 mg/mL (1:3.9) and 1.0 mg/mL (1:1.3) induced significantly higher FcγRIIIA activation in cells infected with gB-N141Q than in cells infected with wild-type HSV-1(F) or gB-N141Q-repair. The gB and gD antibodies present in samples of human γ-globulins at a concentration of 3.04 mg/mL were then depleted by treatment with purified gB-SE or gD-SE (Fig. S7). The anti-gB antibody-depleted human γ-globulins could barely detect gB ectopically expressed by HEK293FT cells (Fig. S7E). In this case, the anti-gB antibody-depleted human γ-globulins (~1.0 mg/mL) induced slightly increased FcγRIIIA-mediated activation in cells infected with gB-N141Q than in cells infected with wild-type HSV-1(F) or gB-N141Q-repair (Fig. 5B), because we used the depleted human γ-globulins at a much higher concentration than the concentration of the depleted human γ-globulins used in the neutralizing assay described in the previous section. However, the degrees of differences between the levels of FcγRIIIA-mediated activation in cells infected with gB-N141Q and the levels of FcγRIIIA-mediated activation in cells infected with wild-type HSV-1(F) or gB-N141Q-repair in cultures containing anti-gB antibody-depleted human γ-globulins were lower than the degrees of differences between the levels of FcγRIIIA-mediated activation in cells infected with those viruses in cultures containing mock-depleted or anti-gD antibody-depleted human γ-globulins (Fig. 5B).

**FIG 6:**
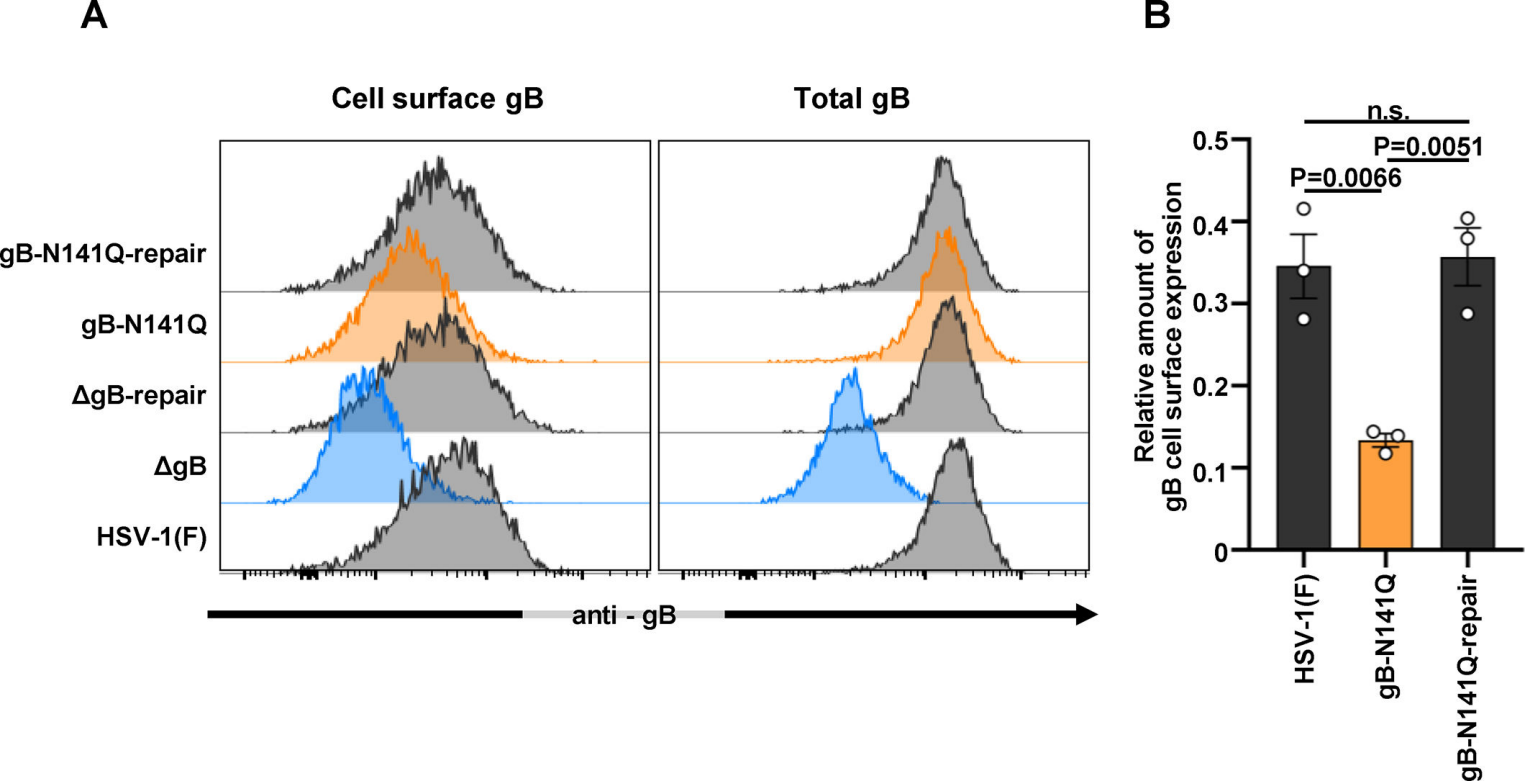
Effect of N-glycan at gB Asn-141 on cell surface expression of gB in HSV-1-infected cells. (A) Vero cells were infected with wild-type HSV-1(F), gB-N141Q, gB-N141Q-repair, ΔgB, or ΔgB-repair at an MOI of 1. At 24 h postinfection, cell surface expression (left panel) and total expression (right panel) of gB in infected cells were measured by flow cytometry. (B) Quantitative bar graph of the cell surface expression of gB shown in (A). The relative amount of expression of gB on the cell surface was calculated as follows: [(mean fluorescent intensity for gB expression on the surfaces of cells infected with the indicated virus) − (mean fluorescent intensity for gB expression on the surfaces of cells infected with ΔgB)]/[(mean fluorescent intensity for total gB expression in cells infected with the indicated virus) − (mean fluorescent intensity for total gB expression in cells infected with ΔgB)]. The data are representative of three independent experiments (A). Each value is the mean ± standard error of the results of three independent experiments (B). Statistical analysis was performed by one-way ANOVA followed by the Tukey test. n.s., not significant (B). ANOVA, analysis of variance.

To eliminate the possibility that the higher level of FcγRIIIA-mediated activation in gB-N141Q-infected cells was due to increased expression of mutated gB on the surface of the infected cells, we investigated the effect of the N-glycan at gB Asn-141 on the expression of gB in the infected cells. Vero cells were infected with wild-type HSV-1(F), gB-N141Q, gB-N141Q-repair, ΔgB, or ΔgB-repair as described in the experiments reported in the previous paragraph and depicted in Fig. 5 and used flow cytometry to show the level of gB expression on the surface of infected cells or the total accumulation of gB in the infected cells. As shown in Fig. 6, the levels of expression of gB on the surface of cells infected with gB-N141Q were significantly lower than those levels on cells infected with wild-type HSV-1(F) or gB-N141Q-repair. In contrast, the total level of gB in cells infected with gB-N141Q was similar to the total levels in cells infected with wild-type HSV-1(F) or gB-N141Q-repair (Fig. 6). These results suggest that the N-glycan at gB Asn-141 was required for the efficient expression of gB on the surface of HSV-1-infected cells. Thus, although cells infected with gB-N141Q expressed lower levels of mutated gB on their surface membranes than the levels of gB expressed by cells infected with wild-type HSV-1(F) or gB-N141Q-repair, human γ-globulins resulted in increased FcγRIIIA-mediated activation of cells infected with gB-N141Q than seen for cells infected with wild-type HSV-1(F) or gB-N141Q-repair. These results eliminated the possibility that the higher level of FcγRIIIA-mediated activation in gB-N141Q-infected cells was due to the increased expression of mutated gB on the surface of the infected cells. Altogether, the results suggest that the N-glycan at gB Asn-141 was required for the efficient evasion of ADCC induced by human antibodies to gB in infected cell cultures.

### Effects of the N-glycan on gB Asn-141 on the replication of HSV-1 in the eyes of mice in the presence of human antibodies

**FIG 7:**
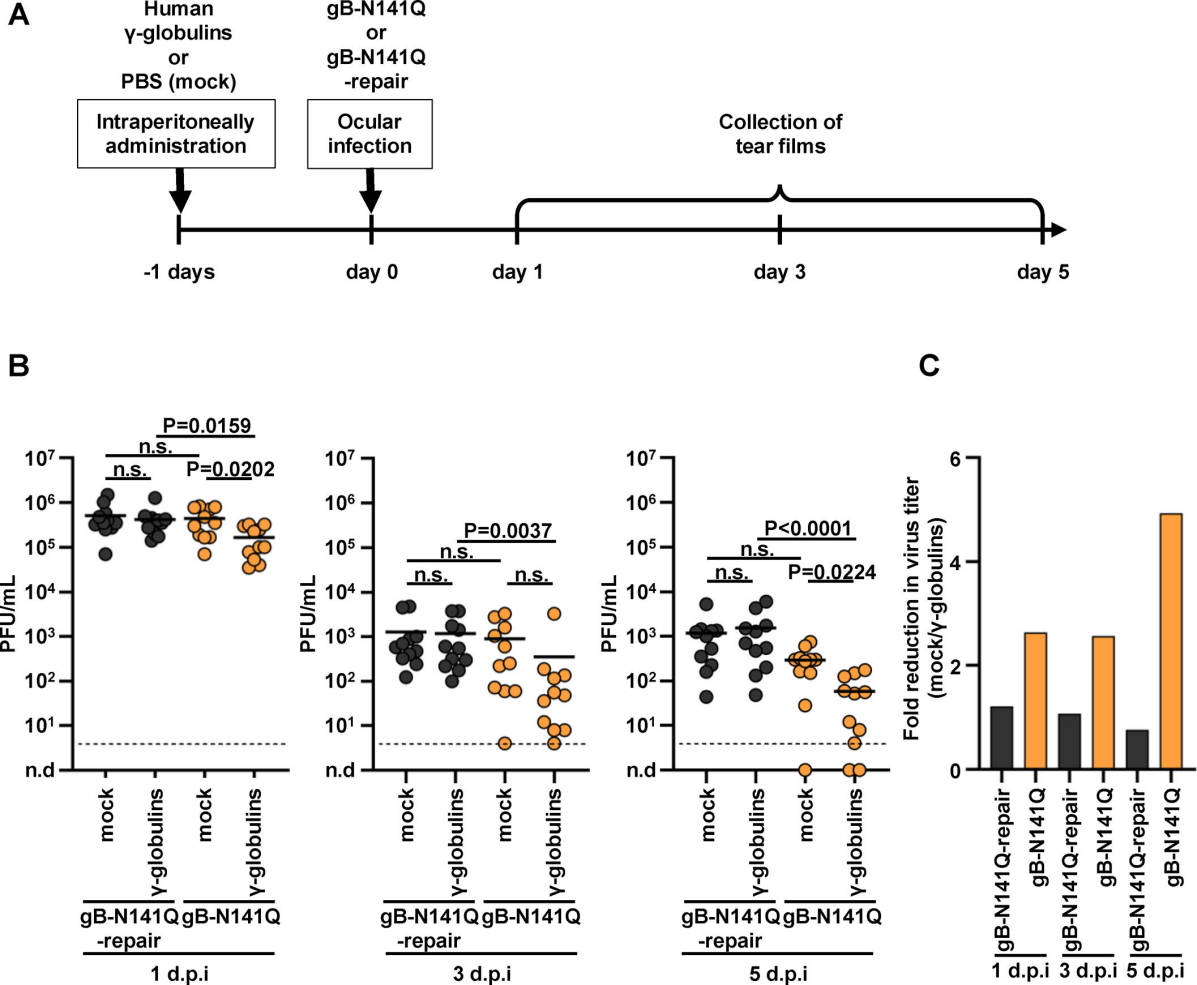
Effect of N-glycan shield at Asn-141 on gB on HSV-1 replication in the eyes of mice in the presence of human γ-globulins. (A) Schematic of the experiment over time. Eleven 4-week-old female mice were mock injected or injected intraperitoneally with human γ-globulins, and 1 day later were then ocularly infected with 3 × 10^6^ PFU/eye of gB-N141Q or gB-N141Q-repair. Samples of tear films were collected at 1, 3, and 5 days postinfection; and viral titers of the tear films were determined. (B) Viral titers of samples collected at each time point. Each data point represents the viral titer of a tear film sample from a single mouse. Horizontal bars indicate the mean for each group. Statistical analysis was performed by one-way ANOVA followed by the Tukey multiple comparisons test. n.s., not significant; d.p.i., days postinfection. Dashed lines indicate limit of detection. n.d., not detected. (C) Fold reduction in mean values of viral titers due to administered human γ-globulins, which are shown in B. ANOVA, analysis of variance.

To examine the effects of human antibodies on HSV-1 replication *in vivo* in the presence or absence of the N-glycan at gB Asn-141, mice were mock injected or injected intraperitoneally with pooled human γ-globulins and were then ocularly infected with gB-N141Q or gB-N141Q-repair 1 day after injection (Fig. 7A). Samples of tear films were collected at the indicated times (Fig. 7A), and viral titers in the tear films were measured. As shown in Fig. 7B, the presence of human γ-globulins did not affect the viral titers of the tear films in mice infected with gB-N141Q-repair at 1, 3, and 5 days postinfection. In contrast, the presence of human γ-globulins significantly reduced viral titers of the tear films of mice infected with gB-N141Q at 1 and 5 days postinfection (Fig. 7B). Thus, the ratios of gB-N141Q titers in the absence of human γ-globulins to those in the presence of human γ-globulins were higher than the ratios of gB-N141Q-repair titers in the absence of human γ-globulins to those in the presence of the human γ-globulins (Fig. 7C). Furthermore, viral titers of the tear films of mice infected with gB-N141Q in the presence of human γ-globulins at 1, 3, and 5 days postinfection were significantly lower than the titers of the tear films of mice infected with gB-N141Q-repair (Fig. 7B). In contrast, the viral titers of the tear films of mice infected with gB-N141Q in the absence of human γ-globulins at 1, 3, and 5 days postinfection were comparable to those of the tear films of mice infected with gB-N141Q-repair; although, as the infection progressed, the viral titers of the tear films of mice infected with gB-N141Q in the absence of human γ-globulins tended to be lower than those titers in mice infected with gB-N141Q-repair. These results indicate that the presence of human antibodies inhibited the replication of gB-N141Q in the peripheral organs of mice more efficiently than it inhibited the replication of gB-N141Q-repair and also suggest that the N-glycan at gB Asn-141 was required for the efficient evasion of HSV-1 from human antibodies *in vivo*. It has been reported that human IgG subclasses could bind to murine Fcγ Rs and efficiently activate murine cells to enable ADCC (29, 30), suggesting that the effects of pooled human γ-globulins in the experiments described in this section might result from both neutralization and ADCC.

### Effects of the N-glycan on gB Asn-141 on the replication of HSV-1 in the eyes of mice immunized against HSV-1

**FIG 8:**
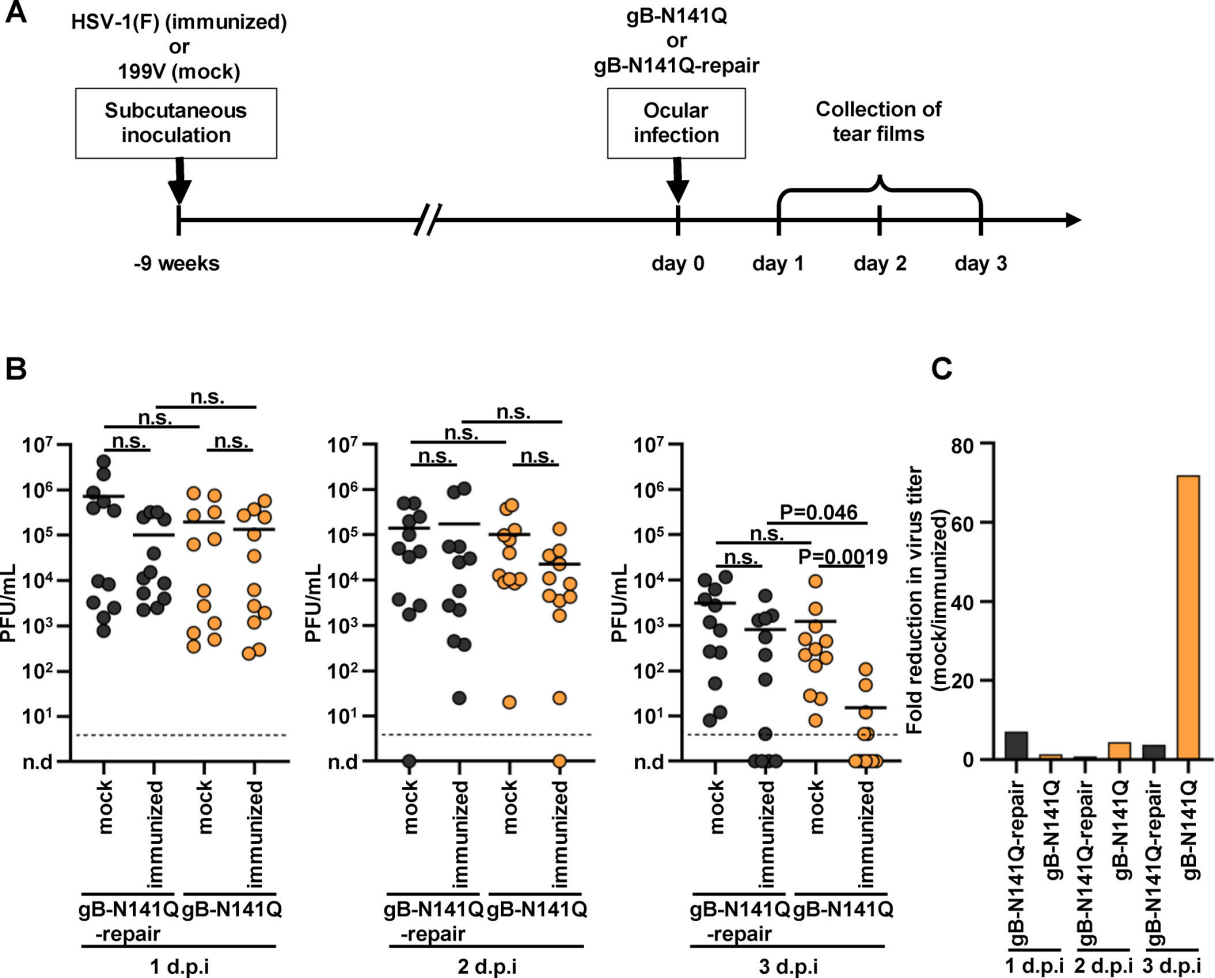
Effects of N-glycan shield at Asn-141 in gB on HSV-1 replication in the eyes of mice immunized with HSV-1. (A) Schematic of the experiment over time. Twelve 3-week-old female mice were subcutaneously mock immunized or immunized with 5 × 10^5^ PFU of wild-type HSV-1(F). At 9 weeks after inoculation, the immunized mice were ocularly infected with 3 × 10^6^ PFU/eye of gB-N141Q or gB-N141Q-repair. Samples of tear films were collected at 1, 2, and 3 days postinfection, and viral titers of the tear films were determined. (B) Viral titers of samples collected at each time point. Each data point represents the viral titer of a tear film sample from a single mouse. Horizontal bars indicate the mean for each group. Statistical analysis was performed by one-way ANOVA followed by the Tukey multiple comparisons test. n.s., not significant; d.p.i., days postinfection. Dashed lines indicate limit of detection. n.d., not detected. (C) Fold reduction in mean values of viral titers due to immunization with HSV-1(F), which is shown in B. ANOVA, analysis of variance.

To examine the effects of the N-glycan at gB Asn-141 on HSV-1 replication *in vivo* in the presence of physiologically induced immunity against HSV-1, mice were subcutaneously mock immunized or immunized with wild-type HSV-1(F). At 9 weeks after inoculation, the immunized mice were ocularly infected with gB-N141Q or gB-N141Q-repair (Fig. 8A). Samples of tear films were collected at the indicated times, and viral titers of the tear films were determined (Fig. 8B). As shown in Fig. 8B, whereas the viral titers of the tear films of immunized mice infected with gB-N141Q at 1 and 2 days postinfection were comparable to those in mock-immunized mice, the viral titers of the tear films of immunized mice infected with gB-N141Q at 3 days postinfection were significantly lower than those in the mock-immunized mice. In contrast, the viral titers of the tear films of immunized mice infected with gB-N141Q-repair at 1, 2, and 3 days postinfection were comparable to those in mock-immunized mice infected with gB-N141Q-repair (Fig. 8B). Thus, the ratios of gB-N141Q titers in mock-immunized mice to those in immunized mice at 3 days postinfection were higher than the ratios of gB-N141Q-repair titers in mock-immunized mice to those in immunized mice (Fig. 8C). Furthermore, the viral titers of the tear films in mock-immunized or immunized mice infected with gB-N141Q at 1 and 2 days postinfection were comparable to those in mock-immunized or immunized mice infected with gB-N141Q-repair (Fig. 8C). In contrast, the viral titers of the tear films in immunized mice infected with gB-N141Q at 3 days postinfection were significantly lower than those in immunized mice infected with gB-N141Q-repair, although viral titers of the tear films in mock-immunized mice infected with gB-N141Q at 3 days postinfection were comparable to those in mock-immunized mice infected with gB-N141Q-repair (Fig. 8B). These results indicate that the presence of immunity against HSV-1 in mice inhibited replication of gB-N141Q more efficiently than the replication of gB-N141Q-repair and suggest that the N-glycan at gB Asn-141 was required for the efficient evasion of HSV-1 from immunity induced in mice previously immunized against HSV-1.

### Effects of the N-glycan at gB Asn-141 on HSV-1 neurovirulence and replication in the CNS of naïve mice

**FIG 9:**
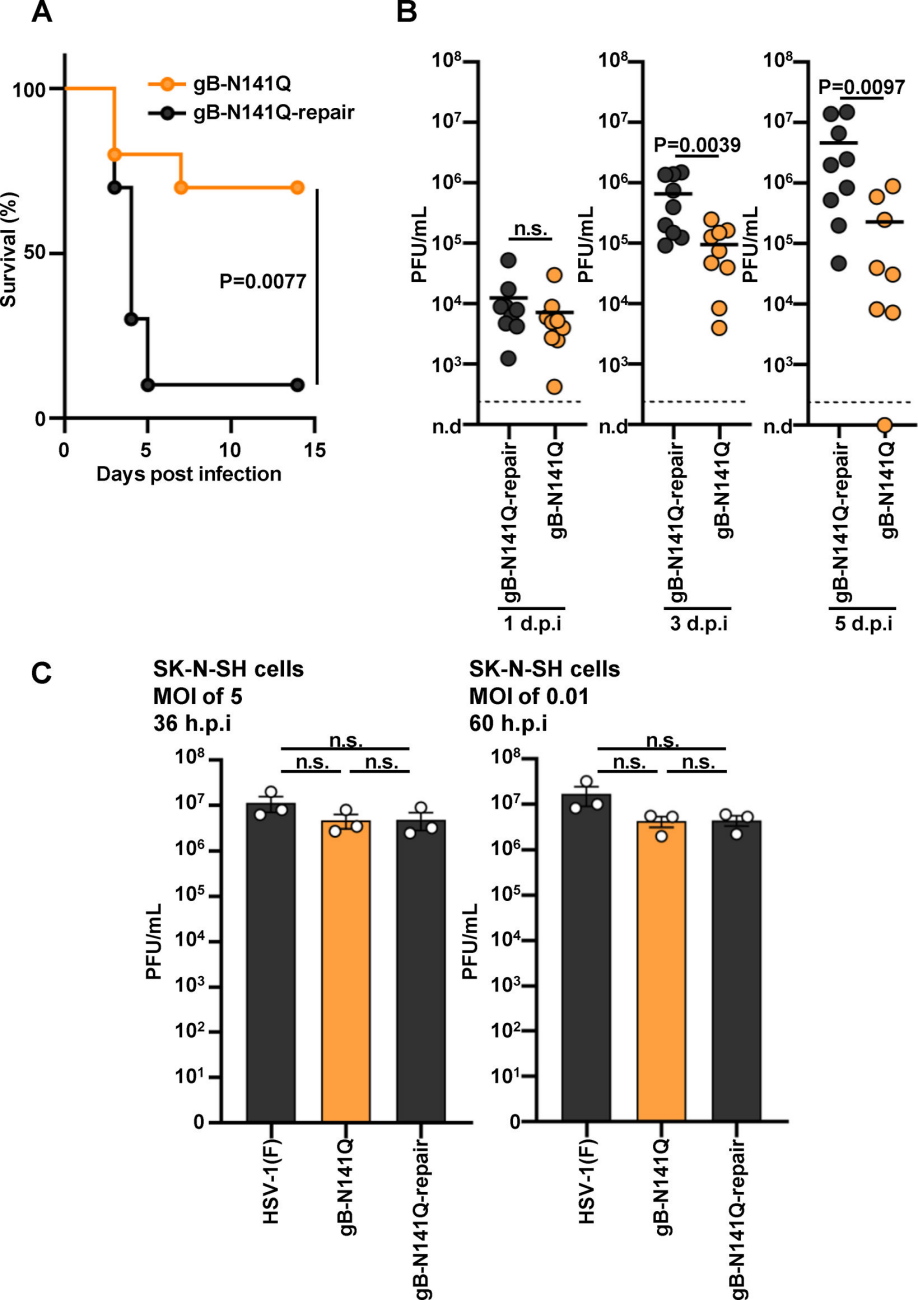
Effects of N-glycan shield at Asn-141 in gB on the neurovirulence of HSV-1 and its replication in the CNS of naïve mice. (A) Ten 3-week-old female mice were inoculated intracranially with 1 × 10^3^ PFU of gB-N141Q or gB-N141Q-repair. Infected mice were monitored for 14 days. (B) Three-week-old female mice were inoculated intracranially with 1 × 10^3^ PFU of gB-N141Q (*n* = 26) or gB-N141Q-repair (*n* = 27). At days 1 (*n* = 9), 3 (*n* = 9), and 5 (gB-N141Q, *n* = 8; gB-N141Q-repair, *n* = 9), mice from each inoculated group were sacrificed, and the viral titers in the brains were determined. Each data point is the viral titer in the brain of a single mouse. Dashed line indicates the limit of detection. n.d., not detected; d.p.i., days postinfection. (C) SK-N-SH cells were infected with wild-type HSV-1(F), gB-N141Q, or gB-N141Q-repair at an MOI of 5 or 0.01. Total viruses from cell culture supernatants and infected cells were harvested at 36 h or 60 h postinfection (p.i.) and assayed on Vero cells. Each value is the mean ± standard error of the results of three independent experiments. Statistical analysis was performed by the log-rank test (A), the two-tailed Student *t* test (B), or one-way ANOVA followed by the Tukey test (C). n.s., not significant; ANOVA, analysis of variance.

To investigate the effects of the N-glycan at gB Asn-141 on the neurovirulence and replication of HSV-1 in the CNS of naïve mice, mice were infected intracranially with gB-N141Q or gB-N141Q-repair, and the mortality rates of these injected mice were monitored for 14 days. As shown in Fig. 9A, the mortality rate of mice infected with gB-N141Q was significantly lower than the rate of mice infected with its repaired virus (gB-N141Q-repair). We also harvested the brains of mice infected with gB-N141Q or gB-N141Q-repair at 1, 3, and 5 days postinfection and measured viral titers in their brains. As shown in Fig. 9B, the viral titers in the brains of mice infected with gB-N141Q at 1 day postinfection were comparable to those of mice infected with gB-N141Q-repair. In contrast, at later time points (3 and 5 days postinfection), viral titers in the brains of mice infected with gB-N141Q were significantly lower than the titers in the brains of mice infected with gB-N141Q-repair. These results suggest that the N-glycan at gB Asn-141 was required for efficient HSV-1 neurovirulence and replication in the CNS of naïve mice. The results also led us to investigate whether the N-glycan at gB Asn-141 acted specifically in neural cells. As shown in Fig. 9C, progeny virus yields in human neuroblastoma SK-N-SH cells infected with gB-N141Q were similar to those in the cells infected with wild-type HSV-1(F) or gB-N141Q-repair. These results further supported our observation from the results of *in vitro* experiments described previously that N-glycosylation on gB does not appear to play a role in the replication of HSV-1 in cell cultures.

## DISCUSSION

It is unquestionable that studies using human samples to analyze the mechanisms of infection utilized by human pathogenic viruses are crucial for understanding the mechanisms effective in humans. Considering that the gap between what can be observed from *in vitro* and *in vivo* viral infections is significant, evaluations of human samples *in vivo* should provide more valuable information on the mechanisms of infection than evaluations of the samples *in vitro*. However, there has been a lack of *in vivo* information because human samples that could be used for *in vivo* analyses as well as *in vivo* models that could represent the pathogenesis of viral infections in humans are limited.

In this study, we clarified a novel immune evasion mechanism used by HSV-1, namely, that an N-glycan shield at Asn 141 of the HSV-1 gB mediated evasion from the deleterious effects of human antibodies such as *in vitro* neutralization and ADCC. A molecular model of N-glycosylation at gB Asn-141, which is based on the prefusion structure of HSV-1 gB (23), predicts that N-glycosylation at gB Asn-141 masks 27 amino acids in the gB molecule (Fig. S8). Of these amino acids, 19 were mapped to the functional region (FR)2 and FR3 of gB (Fig. S8), both of which were previously defined based on the epitopes seen for a panel of neutralizing monoclonal antibodies (31, 32). Notably, the N-glycosylation at gB Asn-141 was predicted to mask Asp-419, a residue critical for the binding of gB to the C226 antibody, which shows high neutralizing activity in preventing the association of gB with a complex of gH and gL and fusion (32). Furthermore, the amino acids predicted to be masked by N-glycosylation at Asn-141 included or were positioned near those (Pro-361, Asp-408, Asp-419, Asn-430, Asn-458, Arg-470, Pro-481, Ile-495, and Thr-497) previously shown to be critical for gB receptor- or gD receptor-mediated fusion (33 - 35), introducing the possibility that antibodies target these amino acid residues. These results suggest that the N-glycan at gB Asn-141 prevented the binding of antibodies to gB epitopes and further support our conclusion that the N-glycan at gB Asn-141 was required for the efficient evasion of HSV-1 from human antibodies. Similarly, HSV-1 envelope glycoproteins, including gC and a complex of gI and gE, have been reported to mediate evasion from human antibodies *in vivo* (36 - 38). The gI/gE complex acts as an Fc receptor that captures human IgG (39, 40). The gC binds to the complement component C3b (41), inhibits complement activation that enhances antibody neutralization (42, 43), and shields gB from complement-independent antibody neutralization (44). Thus, HSV appears to have evolved multiple mechanisms to mediate evasion from human antibodies, highlighting the importance of antibodies for controlling HSV infections in humans as described above.

Based on the structure of gB shown in Fig. 1A, although N-glycans at gB Asn-398, Asn-430, and Asn-489 appear to be adjacent to the N-glycan at gB Asn-141, only this specific N-glycan was shown to shield gB from human γ-globulins. Similar observations were reported in other enveloped viruses (15, 16, 45). We speculate that the N-glycan at gB Asn-141 might cover an area different from the area that the N-glycans at gB Asn-489, Asn-398, and Asn-430 cover; the orientation of the N-glycan at gB Asn-141 might vary or the N-glycan at gB Asn-141 might exhibit different flexibility or different macro- and microheterogeneity, making it unique in its ability to modulate the antigenic effects of gB.

One may argue that the difference between the sensitivities of wild-type HSV-1 and gB-N141Q to neutralization by human γ-globulins was due to slightly less incorporation of the mutant gB into virions. However, it seems unlikely, based on earlier studies of various viruses showing that decreased amounts of expression of a viral antigen by various virions are associated with decreased sensitivities to neutralization (46 - 50).

Our observations that the N-glycan shield on the specific site of HSV-1 gB seemed to significantly increase viral replication in the eyes of mice not only in the presence of HSV-1 immunity but also in the presence of human antibodies, support our prediction from the clarified *in vitro* effects of the glycan shield on HSV-1 that the glycan shield would be effective in the presence of human antibodies *in vivo* and probably in human beings. We should note that the immune response to HSV-1 exhibited by mice immunized by wild-type HSV-1 in this study included not only antibody responses but also a T-cell-mediated immune response and innate immune responses. Interestingly, we also found that the N-glycan shield at a specific site on HSV-1 gB was required for viral neurovirulence and efficient replication in the CNS of naïve mice. Thus, we have identified an important glycan shield on the HSV-1 gB that appears to have two affects: *in vivo* evasion from human antibodies and neurovirulence in naïve hosts. Considering the clinical features of HSV-1 infection, these results suggest that the glycan shield not only facilitates recurrent HSV-1 infections in latently infected humans by evading antibodies but also is important for HSV-1 pathogenesis during the initial infection.

At present, the mechanisms involved in viral neurovirulence and *in vivo* replication that are induced by N-glycosylation at gB Asn-141 remain unclear. It has been reported that gB is a determinant of viral neurovirulence in mice (51) and that the appropriate expression of gB on the cell surface was required for HSV-1 neurovirulence and efficient replication in the CNS of mice (52). Together with the observations in this study that N-glycosylation at gB Asn-141 was required for the efficient expression of gB on the cell surface, the appropriate expression of gB on the cell surface that is regulated by the N-glycosylation of gB at a specific site might be involved in the *in vivo* neurovirulence and replication of HSV-1.

In this study, we used pooled human γ-globulins from human blood as human antibodies. HSV-1 is a ubiquitous human pathogen; approximately 70% of the global human population is infected with HSV-1, and most HSV-1-infected humans have been reported to be latently infected with the virus (1 - 26 - 26). Therefore, it is conceivable that the effects of pooled human γ-globulins represent the effects of antibodies in humans latently infected with HSV-1. Thus, the mouse model with passive transfer of pooled human γ-globulins used in this study potentially mimicked the *in vivo* effects of human antibodies in humans latently infected with HSV-1. HSV-1 frequently reactivates from latent infections and is transmitted to new human hosts. Therefore, the host’s immune responses to HSV-1 persist in latently infected humans because of the repeated stimulation of the immune system, resulting in the progressive enhancement of long-term immunity (1 - 3). The viral strategy of using the N-glycan shield at a specific site of HSV-1 gB to evade antibodies, which was clarified in this study, may protect reactivated viruses from existing antibodies to HSV-1 in latently infected humans and thereby facilitate their transmission to new human hosts. Notably, the N-glycosylation site on HSV-1 gB is widely conserved in viruses subclassified in the alphaherpesvirus subfamily of herpesviruses (53), suggesting that this is a general viral mechanism of evasion from the immune system. Moreover, clarification of the HSV-1 mechanism of evasion from antibodies supports earlier conclusions (5 - 9) that were based on previous clinical trials of HSV vaccines; namely, that antibodies are essential to the control of HSV-1 infections in humans. Additional studies to identify other glycan shields against human antibodies on HSV envelope glycoproteins are important and should be of interest. Those studies and this present study may provide insights into the design of effective therapeutic HSV vaccines against frequent recurrences of herpes virus infections such as genital herpes.

Previous studies have characterized N-glycosylation at Asn-133 of HSV-2 gB and at Asn-154 of the pseudorabies virus (PRV) gB, which correspond to the N-glycosylation at Asn-141 of HSV-1 gB (54, 55). None of those studies addressed the effects of the N-glycosylation of those viruses’ gB on *in vivo* evasion from antibodies and pathogenesis. In agreement with our observation in this study that the N141Q mutation in HSV-1 gB did not affect viral replication in Vero and SK-N-SH cells, the ectopic expression of the PRV gB-N154Q mutant rescued the entry deficiency of a gB-deficient PRV so that its entry would be at a level similar to that of PRVs with wild-type gB (55). In contrast, the ectopic expression of the HSV-2 gB-N133Q mutant barely rescued the entry deficiency of a gB-deficient HSV-2 (54). As observed with the N141Q mutation in HSV-1 gB in the context of viral infection, the ectopic expression of both the HSV-2 gB-N133Q and PRV gB-N154Q mutants showed impaired cell surface expression of the mutants. These observations point out both the similarities in and differences between the roles of the N-glycosylation of gB in viruses.

## MATERIALS AND METHODS

### Cells and viruses

Vero, HEK293FT, Plat-GP, and SK-N-SH cells were described previously (56, 57). Wild-type HSV-1(F) was described previously (58).

### Plasmids

The construction of pFLAG-CMV2-EGFP and pcDNA-MEF-gB was described previously (56, 59). First, to construct pBS-KanR-ePheS*, oligonucleotides making up a kanamycin resistant (KanR) cassette with the I-SceI recognition site and an ePheS* cassette, which has T251A/A294G mutations in ePheS, were amplified from the DNA templates of pEPkan-S (24) and pUC18K ePAG2 (25), respectively. The primers that were used are listed in Table S1B. Then, the two linear DNA fragments were fused by PCR and cloned into the pBluescript KS(+) (Stratagene), as described previously (60). Second, pBS-TEV-2xStrep-KanS was constructed by cloning the kanamycin resistance (KanR) cassette with the I-SceI recognition site, which was amplified by PCR from the pEPkan-S template with the use of primers that additionally encoded the Tobacco Etch Virus (TEV) protease cleavage-site and tandem-strep epitopes as listed in Table S1B, into the pBluescript KS(+). Third, pcDNA3.1-TagRFP-P2A and pcDNA3.1-P2A-TagRFP were constructed by cloning the TagRFP open reading frame (ORF), which was amplified by PCR from pTagRFP-N1 (61) with the use of primers that additionally encoded the 2A self-cleaving peptide fused to its carboxyl terminus (TagRFP-P2A) or amino-terminus (P2A-TagRFP) as listed in Table S1B, respectively, into pcDNA3.1 (Invitrogen, Carlsbad, CA, USA). Fourth, pcDNA3.1-TagRFP-P2A-stop was constructed by cloning annealed DNA oligonucleotides listed in Table S1B into pcDNA3.1-TagRFP-P2A. Fifth, pcDNA3.1-gB-P2A-TagRFP was constructed by cloning gB ORF, which was amplified by PCR from the HSV-1(F) genome isolated as described previously (62), using the primers listed in Table S1B into pcDNA3.1-P2A-TagRFP by the In-Fusion HD Cloning Kit (TaKaRa, Shiga, Japan), according to the manufacturer’s instructions. Sixth, pcDNA3.1-gD-P2A-TagRFP was constructed by cloning gD ORF, which was amplified by PCR from the HSV-1(F) genome using primers listed in Table S1B into pcDNA3.1-P2A-TagRFP by the In-Fusion HD Cloning Kit. Seventh, pFLAG-CMV2-Us6 was constructed by cloning the gD ORF, which was amplified by PCR from pYEbac102 (58) using the primers listed in Table S1B into pFLAG-CMV2 (Sigma-Aldrich, St. Louis, MO, USA).

To construct pRetroX-TRE3G-gBo and pRetroX-TRE3G-ICP4o, the sequences of codon-optimized UL27(gBo) and α4 (ICP4o), which are shown in Table S1C, were engineered according to the GenScript’s OptimumGene algorithm and then synthesized and cloned into pRetroX-TRE3G (TaKaRa) by GenScript.

To generate a fusion protein of maltose binding protein (MBP) and a domain of HSV-2 VP5, pMAL-VP5o-P3 was constructed by amplifying the domains encoding the codon-optimized HSV-2 VP5(VP5o) codons 401 to 600 by PCR from pEGFP-VP5o, which was engineered according to the GenScript’s OptimumGene algorithm, synthesized (Genscript, Piscataway, NJ, USA) using the primers listed in Table S1B, which was followed by cloning the DNA fragments into pMAL-c (New England BioLabs, Ipswich, MA, USA) in frame with MBP. The VP5o sequence is shown in Table S1C.

### Establishment of stable Vero cells with tetracycline-inducible codon-optimized gB and ICP4 (gBo and ICP4o) expression

Vero cells were transduced with supernatants of Plat-GP cells cotransfected with pMDG (63) and pRetroX-Tet3G (TaKaRa), selected with 1  mg/mL G418 solution (Wako, Osaka, Japan) to generate Tet3G-Vero cells. The cells were further transduced with a mixture of supernatants of Plat-GP cells cotransfected with pMDG and pRetroX-TRE3G-gBo, and supernatants of Plat-GP cells co-transfected with pMDG and pRetroX-TRE3G-ICP4o to establish gBo/ICP4o-TetON-Vero cells. After double selection with 1 mg/mL of G418 solution and 5 µg/mL of puromycin, a single clone in which expression of gBo and ICP4o was induced by DOX was selected.

### Two-step Red-mediated recombination using the KanR/ePheS* cassette

The two-step Red-mediated mutagenesis procedure used in this study was performed as described previously (24, 64). Briefly, linear DNA fragments containing an I-SceI recognition sequence, KanR and ePheS*cassettes, and target homologous sequences were amplified by PCR from pBS-KanR-ePheS* using the primers listed in Table S1D. The linear fragments were electroporated into the electrocompetent *E. coli* strain GS1783 containing the pYEbac102Cre (58, 65). The transformed bacteria were then incubated at 32℃ for 40 to 60 min and plated on LB agar plates containing 20 µg/mL of chloramphenicol and 40 µg/mL of kanamycin to select *E. coli* clones harboring pYEbac102Cre containing the KanR and ePheS* cassettes (KanR/ePheS* cassettes). Kanamycin-resistant colonies were screened by PCR with the appropriate primers. Next, the KanR/ePheS* cassettes were excised by expressing the I-SceI homing enzyme in GS1783 through induction with arabinose, followed by induction of the Red recombination machinery by raising the temperature. Briefly, 100 µL of an overnight culture of kanamycin-resistant *E. coli* clones grown in LB medium containing chloramphenicol and kanamycin was inoculated into 2 mL of LB medium containing chloramphenicol only. Bacteria were incubated at 32°C for 2–4 h with shaking, followed by addition of 10% (wt/vol) L-arabinose (Wako) to the culture at a 1:5 ratio, and incubated for another 1 h at 32°C. Finally, the *E. coli* culture was incubated at 42°C for 30 min. It was then shaken at 32°C for another 1–2 h, and 50 µL of 10^−3^ to 10^−4^ dilutions of the culture was plated onto LB agar plates containing 20 µg/mL of chloramphenicol and 1 mM of 4-CP to select *E. coli* clones harboring the pYEbac102Cre, from which the KanR/ePheS* cassette was excised. Chloramphenicol- and 4-CP-resistant colonies were screened by PCR with appropriate primers, which was followed by nucleotide sequencing for confirmation of the desired mutation.

### Generation of recombinant HSV-1

Recombinant viruses YK650 (UL51-T190A_KanR/ePheS*), YK681 (gB-N87Q), YK683 (gB-N141Q), YK685 (gB-N398Q), YK687 (gB-N430Q), YK689 (gB-N489Q), YK691 (gB-N674Q), YK693 (gB-N888Q), YK682 (gB-N87Q-repair), YK684 (gB-N141Q-repair), YK686 (gB-N398Q-repair), YK688 (gB-N430Q-repair), YK690 (gB-N489Q-repair), YK692 (gB-N674Q-repair), and YK694 (gB-N888Q-repair) (Fig. 1) were generated by the two-step Red-mediated mutagenesis procedure using the KanR/ePheS* cassette as described in the previous section with the primers listed in Table S1D. The recombinant virus YK649 (UL51-T190A_KanR) was generated by the two-step Red-mediated mutagenesis procedure using *E. coli* GS1783 containing pYEbac102Cre, as described previously (24, 64), with the exception that the primers used instead of those described previously are listed in Table S1D. The recombinant virus YK695 (ΔgB), in which the UL27 gene encoding gB was disrupted by deleting gB codons 1–727 with a kanamycin resistance gene, was generated by the two-step Red-mediated mutagenesis procedure using *E. coli* GS1783 containing pYEbac102Cre, as described previously (24, 64), with the exception that the primers used instead of those described previously are listed in Table S1D.

The recombinant virus YK696 (ΔgB-repair), in which the deletion mutation in gB was repaired, was generated by cotransfection with pYEbac102Cre carrying the gB-deletion mutation and pCRxgB (66) into Vero cells. Plaques were isolated and purified on Vero cells. Restoration was confirmed by nucleotide sequencing.

The recombinant virus YK717 (gB-SE), which expresses gB fused to a TEV protease cleavage site and a Strep-tag; and recombinant virus YK718 (gD-SE), which expresses gD fused to a TEV protease cleavage site and a Strep-tag, were generated by the two-step Red-mediated mutagenesis procedure using *E. coli* GS1783 containing pYEbac102Cre, as described previously (24, 64), with the exception that the primers used instead of those described previously are listed in Table S1D.

In experiments in which YK695 (HSV-1 ΔgB) was used, viruses were propagated and assayed in HSV-1 gBo/ICP4o-TetON-Vero cells in the presence of doxycycline (DOX) (1  mg/mL). Other viruses used in this study were propagated and titrated in Vero cells.

### Production and purification of MBP in *E. coli*

The MBP fusion protein MBP-VP5o-P3 was expressed in *E. coli* BL21 Star (DE3) (Thermo Fisher Scientific) that had been transformed with pMAL-VP5o-P3 and purified using amylose resin (New England BioLabs) as described previously (67).

### Antibodies

Commercial antibodies used in this study were mouse monoclonal antibodies to ICP4 (58S; ATCC, Manassas, VA, USA), gB (H1817; Virusys, Taneytown, MD, USA), gD (DL6; Santa Cruz Biotechnology, Dallas, TX, USA), gC (H1A022; Virusys), gH (52 S; ATCC), gL (H1A259; Virusys), α-tubulin (DM1A; Sigma-Aldrich), and ICP8 (MAB8671-I); and rabbit polyclonal antibodies to VP23 (CAC-CT-HSV-UL18; Cosmo Bio, Tokyo, Japan).

To generate rabbit polyclonal antibodies to VP5, rabbits were immunized with purified MBP-VP5o-P3, following the standard protocol of Scrum (Tokyo, Japan).

### PNGase F digestion and immunoblotting

Vero cells were infected with each of the indicated viruses at an MOI of 5 for 21 h and lysed with T-PER Tissue Protein Extraction Reagent (Thermo Scientific, Waltham, MA, USA). The lysates were sonicated and denatured with Glycoprotein Denaturing Buffer (NEB) by heating them at 100°C for 10 min. Aliquots of the lysates were incubated with 2,500 units of PNGase F (NEB) at 37°C for 1 h. Aliquots of the lysates incubated under the same conditions without PNGase F were used as controls. The incubated mixtures were subjected to immunoblotting as described previously (68).

### Detection of gB- or gD-specific antibodies in pooled human γ-globulins by flow cytometry

Pei Max (Polyscience, Inc., Warrington, PA, USA) was used to transfect HEK293FT cells with selected plasmids. At 48 h posttransfection, the transfected cells were detached from their culture plates and washed once with phosphate-buffered saline (PBS) supplemented with 2% fetal calf serum (FCS) (washing buffer). Cells were fixed and permeabilized with Cytofix/Cytoperm (Becton Dickinson, Franklin Lakes, NJ, USA) and incubated with diluted human γ-globulins (G4386; Sigma-Aldrich) on ice for 30 min. After the cells were washed with washing buffer, they were further incubated with anti-human IgG conjugated to Alexa Flour 647 (Invitrogen) on ice for 30 min. After the cells were washed again, they were analyzed with a CytoFLEX S flow cytometer (Beckman Coulter, Brea, CA, USA). The data were analyzed with FlowJo 10.8.1 software (Becton Dickinson).

### Depletion of gB- or gD-specific antibodies from pooled human γ-globulins

Vero cells (5 × 10^7^) were infected with HSV-1(F), gB-SE, or gD-SE at an MOI of 0.5 for 24 h and lysed in 5 mL of radioimmunoprecipitation assay (RIPA) buffer [10 mM Tris-HCl (pH 7.4), 150 mM NaCl, 1% Nonidet P-40 (NP40), 0.1% deoxycholate, 0.1% sodium deodecyl sulfate, and 1 mM EDTA] containing a protease inhibitor cocktail (Nacalai Tesque, Kyoto, Japan). After centrifugation, the supernatants were precleared by incubating with protein G-Sepharose beads (GE Healthcare, Chicago, IL, USA) and reacted with 100 µL of Strep-Tactin Sepharose beads (IBA Lifesciences, Göttingen, Germany) for 4 h at 4°C. The beads were collected by brief centrifugation and washed four times with RIPA buffer and two times with PBS. For the neutralization assay, samples of gB-SE or gD-SE immobilized on Strep-Tactin Sepharose beads were incubated with 1 mL of diluted human γ-globulins (G4386; Sigma-Aldrich) (0.082 mg/mL in medium 199 containing 1% FCS) at 4°C overnight; and after centrifugation, the supernatants containing gB- or gD-antibody depleted human γ-globulins were filtered.

For the ADCC assay, human γ-globulins were incubated twice with gB-SE-beads, gD-SE-beads, or control beads, as follows: samples of gB-SE or gD-SE immobilized on Strep-Tactin Sepharose beads were incubated with 1 mL of diluted human γ-globulins (G4386; Sigma-Aldrich) (3.04 mg/mL in ADCC assay buffer) at 4°C overnight, and after centrifugation, the supernatants were further incubated with gB-SE or gD-SE immobilized on Strep-Tactin Sepharose beads. After centrifugation, the supernatants containing gB- or gD-antibody depleted human γ-globulins were filtered.

Similarly, HSV-1(F)-infected Vero cell lysates prepared as described for the depleted human γ-globulins was incubated with Strep-Tactin Sepharose beads, and after centrifugation and washing, the beads (control beads) were incubated once or twice with human γ-globulins diluted as described, and then centrifuged, followed by filtration of the supernatants to produce samples of mock-depleted human γ-globulins.

### Neutralization assay

Pooled human γ-globulins (G4386; Sigma-Aldrich) or human serum (H4522; Sigma-Aldrich) without heat treatment at 56°C were serially diluted in medium 199 containing 1% FCS. The serial dilutions were mixed 1:1 with 100 PFU of each selected virus in medium 199 containing 1% FCS, incubated at 37°C for 1 h and then inoculated onto Vero cell monolayers to perform plaque assays. At 2 days postinfection, the plaques were counted. The percentage of neutralization was determined as follows: the numbers of plaques formed by the virus samples that had been incubated with or without human γ-globulins as a value with the following formula: 100 × [1− (numbers of plaques produced after incubation of viral samples with human γ-globulins)/(numbers of plaques produced after incubation of viral samples without human γ-globulins)]. Human serum which had been preincubated at 56°C for 30 min to inactivate complement was also used.

### Determination of plaque size

Vero cells were infected with 200 PFU of wild-type HSV-1(F) or each of the recombinant viruses. After adsorption for 1  h, the inoculum was removed, and the cell monolayers were overlaid with medium 199 containing 1% FCS and 1% agarose. At 2  days postinfection, the agarose-containing medium was removed. Cells were then fixed in methanol, permeabilized with 0.1% Triton X-100 in PBS, blocked with 10% human serum (Sigma-Aldrich) in PBS, reacted with anti-ICP4 antibodies, reacted with goat anti-mouse IgG-Alexa Fluor 488 (Invitrogen). Plaques in each culture were examined under a fluorescence microscope equipped with a BZ-X800 (KEYENCE, Osaka, Japan) and BZ-X800 Analyzer (KEYENCE).

### ADCC reporter assay

The extent of ADCC activation induced by human γ-globulins was evaluated with the use of an ADCC Reporter Bioassay (Core Kit; Promega, G7010) and the EnSpireMultimode Plate Reader (PerkinElmer, Waltham, MA, USA). The assay was performed according to the manufacturer’s instructions. Briefly, Vero cells were infected at an MOI of 1 with each selected virus in medium 199 containing 1% FCS. After adsorption for 1  h, the inoculum was removed, and the cell monolayers were overlaid with medium 199 containing 10% FCS. At 24 h postinfection, the culture medium was replaced with ADCC assay buffer containing effector cells and diluted human γ-globulins at a 2:1 ratio and incubated at 37°C for 6 h. Bio-Glo luciferase reagent was then added, and the luciferase signals were quantitated as relative light units (RLUs) on an EnSpire reader. Extent of induction was calculated as follows: Fold induction = (RLUs_with antibody_ − RLUs_background_)/(RLUs_no antibody_ − RLUs_background_).

### Determination of gB expression on the surfaces of HSV-1-infected cells

The expression of HSV-1 glycoproteins on the surfaces of infected cells was analyzed as described previously (52). Briefly, Vero cells were infected at an MOI of 1 with each selected virus in medium 199 containing 1% FCS. After adsorption for 1  h, the inoculum was removed, and the cell monolayers were overlaid with medium 199 containing 10% FCS. At 24 h postinfection, cell monolayers were detached with PBS containing 0.02% EDTA and were washed one time with PBS supplemented with 2% FCS (washing buffer). To analyze the total expression of gB, infected Vero cells were detached as described, and fixed and permeabilized with Cytofix/Cytoperm Fixation/Permeabilization Solution (Becton Dickinson). Treated and untreated cells were then incubated with mouse anti-gB monoclonal antibody in washing buffer on ice for 30 min. After the cells were washed with washing buffer, they were further incubated with anti-mouse IgG conjugated to Alexa Flour 647 dye (Invitrogen) on ice for 30 min. After the cells were washed again, they were analyzed with a CytoFLEX S flow cytometer (Beckman Coulter). The data were analyzed by FlowJo 10.8.1 software (Becton Dickinson).

### Immunofluorescence assays

Immunofluorescence assays were performed as described previously (69).

### Purification of virions

Virions were purified as described previously (70).

### Animal studies

Female ICR mice were purchased from Charles River Laboratories. For ocular infections by each selected virus in mice in the presence of pooled human γ-globulins, 4-week-old mice were injected intraperitoneally with 1,250 mg/kg of human γ-globulins or PBS. One day after administration, the mice were ocularly infected with 3 × 10^6^ PFU/eye of each selected virus, as described previously (57). For mice immunized with HSV-1 before ocular infection, 3-week-old mice were injected subcutaneously in the neck with 5 × 10^5^ PFU of HSV-1(F). The immunized mice were then infected ocularly 9 weeks after immunization with 3 × 10^6^ PFU/eye of each selected virus as described previously (57). Virus titers in the tear films of mice were determined as described previously (71).

For intracranial infections, 3-week-old mice were inoculated intracranially with each selected virus as described previously (57). Mice were monitored daily, and mortality occurring from 1 to 14 days postinfection was attributed to the infecting virus. To measure viral titers in the brains of infected mice, 3-week-old female ICR mice were each inoculated intracranially with 1 × 10^3^ PFU of each selected virus. At 1, 3, and 5 days postinfection, the brains of the mice were harvested, and virus titers were determined on Vero cells. All animal experiments were carried out in accordance with the Guidelines for Proper Conduct of Animal Experiments, Science Council of Japan. The protocol was approved by the Institutional Animal Care and Use Committee (IACUC) of the Institute of Medical Science, the University of Tokyo (IACUC protocol approval number: A21-55).

### Modeling of the N-glycosylated gB protein

The N-glycan core (Man_3_GlcNAc_2_) was modeled according to the prefusion structure of HSV-1 (23) gB (PDB ID: 6Z9M) using the Glycan Reader and Modeler (72) and the CHARMM-GUI program (73, 74). Discovery Studio 2021 software (Dassault Systèmes) was used to change the χ1 angle (N-Cα-Cβ-Cγ) of the Asn141 B chain (PDB ID: 6Z9M) from −178° to −66° to avoid steric clash of the N-glycan with the neighboring polypeptide. Visualization of the protein 3D structure was performed in the PyMol Molecular Graphics System, version 2.5 (Schrödinger, LLC, New York, NY, USA).

### Analysis of protein surface

The AREAIMOL program (CCP4 package, version 6.2) was used to determine the accessible surface area (ASA) (75). A spherical probe with a radius of 10 Å, which is similar to the dimension of the antigen-binding fragments (single-chain variable fragment) of the antibodies, was used in the estimation of the ASA (76, 77). The extent of glycan shielding (ΔASA) was estimated for each amino acid residue by calculating the difference between the ASAs of N-glycosylated and nonglycosylated gB structures [ΔASA = ASA (nonglycosylated gB) − ASA (N-glycosylated gB)].

### Statistical analysis

The unpaired *t* test was used to compare two groups. One-way or two-way ANOVA followed by the Tukey or Dunnett multiple comparisons tests was used for multiple comparisons. A *P* value <0.05 was considered significant. For the statistical analysis of viral titers, data were converted to common logarithms (log_10_). For values below the detection limit, statistical processing was performed assuming that the values were those of the detection limit. GraphPad Prism 8 (GraphPad Software) was used to perform statistical analysis.

## SUPPLEMENTAL MATERIAL

Fig. S1 mbio.00992-23-s0001.tif Schematic diagrams of wild-type HSV-1(F) and the creation of its recombinants that were used in this study.

Fig. S2 mbio.00992-23-s0002.tif Flow chart of two-step Red-mediated recombination using the KanR or KanR/ePheS* cassette.

Fig. S3 mbio.00992-23-s0003.tif Characterization of recombinant HSV-1 generated with the use of the improved HSV-1 genetic manipulation system.

Fig. S4 mbio.00992-23-s0004.tif Effect of mutation in each of the gB N-glycosylation sites on HSV-1 replication in cell cultures.

Fig. S5 mbio.00992-23-s0005.tif Detection of gB and gD antibodies in human γ-globulins.

Fig. S6 mbio.00992-23-s0006.tif Effect of mutation in each of the gB N-glycosylation sites on viral susceptibility to neutralization by human γ-globulins.

Fig. S7 mbio.00992-23-s0007.tif Depletion of gB or gD antibodies from pooled human γ-globulins.

Fig. S8 mbio.00992-23-s0008.tif Effects of N-glycan shield at Asn-141 in gB on the antigenicity of gB in the prefusion state.

Table S1 mbio.00992-23-s0009.docx Efficiency of two-step Red-mediated recombination and information on oligonucleotides.

ASM does not own the copyrights to Supplemental Material that may be linked to, or accessed through, an article. The authors have granted ASM a non-exclusive, world-wide license to publish the Supplemental Material files. Please contact the corresponding author directly for reuse.

## References

[1] Knipe DM , Heldwein EE , Mohr IJ , Sodroski CN , Whitley RJ , Johnston C . 2022. Herpes Simplex viruses: Mechanisms of Lytic and latent infection, p 235–296. In Howley PM , DM Knipe , B Damania , JI Cohen , SPJ Whelan , EO Freed , L Enquist (ed), Fields Virology, 7th ed. Lippincott-Williams & Wilkins, Philadelphia, PA.

[2] Roizman B , Knipe DM , Whitley RJ . 2013. Herpes Simplex viruses, p 1823–1897. In Knipe DM , PM Howley , JI Cohen , DE Griffin , RA Lamb , MA Martin , VR Racaniello , B Roizman (ed), Lippincott-Williams & Wilkins. Philadelphia, PA.

[3] Knipe DM , Heldwein EE , Mohr IJ , Sodroski CN , Whitley RJ , Johnston C . 2022. Herpes simplex viruses: pathogenesis and clinical insights, p 297–323. In Howley PM , DM Knipe , B Damania , JI Cohen , SPJ Whelan , EO Freed , L Enquist (ed), Fields virology, 7th ed. Lippincott-Williams & Wilkins, Philadelphia, PA.

[4] Krishnan R , Stuart PM . 2021. Developments in vaccination for herpes simplex virus. Front Microbiol 12:798927. doi:10.3389/fmicb.2021.798927 34950127 PMC8691362

[5] Awasthi S , Friedman HM . 2022. An mRNA vaccine to prevent genital herpes. Transl Res 242:56–65. doi:10.1016/j.trsl.2021.12.006 34954087 PMC8695322

[6] Belshe Robert B , Leone PA , Bernstein DI , Wald A , Levin MJ , Stapleton JT , Gorfinkel I , Morrow RLA , Ewell MG , Stokes-Riner A , Dubin G , Heineman TC , Schulte JM , Deal CD , Herpevac Trial for Women . 2012. Efficacy results of a trial of a herpes simplex vaccine. N Engl J Med 366:34–43. doi:10.1056/NEJMoa1103151 22216840 PMC3287348

[7] Belshe RB , Heineman TC , Bernstein DI , Bellamy AR , Ewell M , van der Most R , Deal CD . 2014. Correlate of immune protection against HSV-1 genital disease in vaccinated women. J Infect Dis 209:828–836. doi:10.1093/infdis/jit651 24285844 PMC3935479

[8] Awasthi S , Belshe RB , Friedman HM . 2014. Better neutralization of herpes simplex virus type 1 (HSV-1) than HSV-2 by antibody from recipients of glaxosmithkline HSV-2 glycoprotein D2 subunit vaccine. J Infect Dis 210:571–575. doi:10.1093/infdis/jiu177 24652496 PMC4172040

[9] Kohl S , West MS , Prober CG , Sullender WM , Loo LS , Arvin AM . 1989. Neonatal antibody-dependent cellular cytotoxic antibody levels are associated with the clinical presentation of neonatal herpes simplex virus infection. J Infect Dis 160:770–776. doi:10.1093/infdis/160.5.770 2553825

[10] Mahant AM , Guerguis S , Blevins TP , Cheshenko N , Gao W , Anastos K , Belshe RB , Herold BC . 2022. Failure of herpes simplex virus glycoprotein D antibodies to elicit antibody-dependent cell-mediated cytotoxicity: implications for future vaccines. J Infect Dis 226:1489–1498. doi:10.1093/infdis/jiac284 35834278 PMC10205893

[11] Vigerust DJ , Shepherd VL . 2007. Virus glycosylation: role in virulence and immune interactions. Trends Microbiol 15:211–218. doi:10.1016/j.tim.2007.03.003 17398101 PMC7127133

[12] Skehel JJ , Stevens DJ , Daniels RS , Douglas AR , Knossow M , Wilson IA , Wiley DC . 1984. A carbohydrate side chain on Hemagglutinins of Hong Kong influenza viruses inhibits recognition by a monoclonal antibody. Proc Natl Acad Sci U S A 81:1779–1783. doi:10.1073/pnas.81.6.1779 6584912 PMC345004

[13] Back NK , Smit L , De Jong JJ , Keulen W , Schutten M , Goudsmit J , Tersmette M . 1994. An N-glycan within the human immunodeficiency virus type 1 gp120 V3 loop affects virus neutralization. Virology 199:431–438. doi:10.1006/viro.1994.1141 8122371

[14] Aguilar HC , Matreyek KA , Filone CM , Hashimi ST , Levroney EL , Negrete OA , Bertolotti-Ciarlet A , Choi DY , McHardy I , Fulcher JA , Su SV , Wolf MC , Kohatsu L , Baum LG , Lee B . 2006. N-glycans on Nipah virus fusion protein protect against neutralization but reduce membrane fusion and viral entry. J Virol 80:4878–4889. doi:10.1128/JVI.80.10.4878-4889.2006 16641279 PMC1472062

[15] Falkowska E , Kajumo F , Garcia E , Reinus J , Dragic T . 2007. Hepatitis C virus envelope glycoprotein E2 glycans modulate entry, Cd81 binding, and neutralization. J Virol 81:8072–8079. doi:10.1128/JVI.00459-07 17507469 PMC1951298

[16] Helle F , Goffard A , Morel V , Duverlie G , McKeating J , Keck ZY , Foung S , Penin F , Dubuisson J , Voisset C . 2007. The neutralizing activity of anti-hepatitis C virus antibodies is modulated by specific glycans on the E2 envelope protein. J Virol 81:8101–8111. doi:10.1128/JVI.00127-07 17522218 PMC1951279

[17] Lennemann NJ , Rhein BA , Ndungo E , Chandran K , Qiu X , Maury W . 2014. Comprehensive functional analysis of N-linked glycans on Ebola virus Gp1. mBio 5:e00862-13. doi:10.1128/mBio.00862-13 24473128 PMC3950510

[18] Julithe R , Abou-Jaoudé G , Sureau C . 2014. Modification of the hepatitis B virus envelope protein glycosylation pattern interferes with secretion of viral particles, infectivity, and susceptibility to neutralizing antibodies. J Virol 88:9049–9059. doi:10.1128/JVI.01161-14 24899172 PMC4136284

[19] Sommerstein R , Flatz L , Remy MM , Malinge P , Magistrelli G , Fischer N , Sahin M , Bergthaler A , Igonet S , ter Meulen J , Rigo D , Meda P , Rabah N , Coutard B , Bowden TA , Lambert P-H , Siegrist C-A , Pinschewer DD , Pierson TC . 2015. Arenavirus glycan shield promotes neutralizing antibody evasion and protracted infection. PLoS Pathog 11:e1005276. doi:10.1371/journal.ppat.1005276 26587982 PMC4654586

[20] Ansari IH , Kwon B , Osorio FA , Pattnaik AK . 2006. Influence of N-linked glycosylation of porcine reproductive and respiratory syndrome virus Gp5 on virus infectivity, antigenicity, and ability to induce neutralizing antibodies. J Virol 80:3994–4004. doi:10.1128/JVI.80.8.3994-4004.2006 16571816 PMC1440468

[21] Truong NR , Smith JB , Sandgren KJ , Cunningham AL . 2019. Mechanisms of immune control of mucosal HSV infection: A guide to rational vaccine design. Front Immunol 10:373. doi:10.3389/fimmu.2019.00373 30894859 PMC6414784

[22] Connolly SA , Jardetzky TS , Longnecker R . 2021. The structural basis of herpesvirus entry. Nat Rev Microbiol 19:110–121. doi:10.1038/s41579-020-00448-w 33087881 PMC8579738

[23] Vollmer B , Pražák V , Vasishtan D , Jefferys EE , Hernandez-Duran A , Vallbracht M , Klupp BG , Mettenleiter TC , Backovic M , Rey FA , Topf M , Grünewald K . 2020. The Prefusion structure of herpes simplex virus glycoprotein B. Sci Adv 6:eabc1726. doi:10.1126/sciadv.abc1726 32978151 PMC7518877

[24] Tischer BK , von Einem J , Kaufer B , Osterrieder N . 2006. Two-step red-mediated recombination for versatile high-efficiency markerless DNA manipulation in Escherichia coli. Biotechniques 40:191–197. doi:10.2144/000112096 16526409

[25] Miyazaki K . 2015. Molecular engineering of a PheS counterselection marker for improved operating efficiency in Escherichia coli. Biotechniques 58:86–88. doi:10.2144/000114257 25652032

[26] James C , Harfouche M , Welton NJ , Turner KM , Abu-Raddad LJ , Gottlieb SL , Looker KJ . 2020. Herpes simplex virus: global infection prevalence and incidence estimates, 2016. Bull World Health Organ 98:315–329. doi:10.2471/BLT.19.237149 32514197 PMC7265941

[27] Erlich KS , Dix RD , Mills J . 1987. Prevention and treatment of experimental herpes simplex virus encephalitis with human immune serum globulin. Antimicrob Agents Chemother 31:1006–1009. doi:10.1128/AAC.31.7.1006 2821882 PMC174861

[28] Kohl S , Strynadka NC , Hodges RS , Pereira L . 1990. Analysis of the role of antibody-dependent cellular cytotoxic antibody activity in murine neonatal herpes simplex virus infection with antibodies to synthetic peptides of glycoprotein D and monoclonal antibodies to glycoprotein B. J Clin Invest 86:273–278. doi:10.1172/JCI114695 2164044 PMC296717

[29] Dekkers G , Bentlage AEH , Stegmann TC , Howie HL , Lissenberg-Thunnissen S , Zimring J , Rispens T , Vidarsson G . 2017. Affinity of human IgG Subclasses to mouse Fc gamma receptors. MAbs 9:767–773. doi:10.1080/19420862.2017.1323159 28463043 PMC5524164

[30] Overdijk MB , Verploegen S , Ortiz Buijsse A , Vink T , Leusen JHW , Bleeker WK , Parren PWHI . 2012. Crosstalk between human IgG isotypes and murine effector cells. J Immunol 189:3430–3438. doi:10.4049/jimmunol.1200356 22956577

[31] Bender FC , Samanta M , Heldwein EE , de Leon MP , Bilman E , Lou H , Whitbeck JC , Eisenberg RJ , Cohen GH . 2007. Antigenic and mutational analyses of herpes simplex virus glycoprotein B reveal four functional regions. J Virol 81:3827–3841. doi:10.1128/JVI.02710-06 17267495 PMC1866100

[32] Atanasiu D , Whitbeck JC , de Leon MP , Lou H , Hannah BP , Cohen GH , Eisenberg RJ . 2010. Bimolecular complementation defines functional regions of herpes simplex virus gB that are involved with gH/gL as a necessary step leading to cell fusion. J Virol 84:3825–3834. doi:10.1128/JVI.02687-09 20130048 PMC2849501

[33] Lin E , Spear PG . 2007. Random linker-insertion mutagenesis to identify functional domains of herpes simplex virus type 1 glycoprotein B. Proc Natl Acad Sci U S A 104:13140–13145. doi:10.1073/pnas.0705926104 17666526 PMC1941792

[34] Fan Q , Lin E , Satoh T , Arase H , Spear PG . 2009. Differential effects on cell fusion activity of mutations in herpes simplex virus 1 glycoprotein B (gB) dependent on whether a gD receptor or a gB receptor is overexpressed. J Virol 83:7384–7390. doi:10.1128/JVI.00087-09 19457990 PMC2708615

[35] Gallagher JR , Atanasiu D , Saw WT , Paradisgarten MJ , Whitbeck JC , Eisenberg RJ , Cohen GH , Longnecker R . 2014. Functional fluorescent protein insertions in herpes simplex virus gB report on gB conformation before and after execution of membrane fusion. PLoS Pathog 10:e1004373. doi:10.1371/journal.ppat.1004373 25233449 PMC4169481

[36] Nagashunmugam T , Lubinski J , Wang L , Goldstein LT , Weeks BS , Sundaresan P , Kang EH , Dubin G , Friedman HM . 1998. In vivo immune evasion mediated by the herpes simplex virus type 1 immunoglobulin G Fc receptor. J Virol 72:5351–5359. doi:10.1128/JVI.72.7.5351-5359.1998 9620988 PMC110157

[37] Lubinski JM , Jiang M , Hook L , Chang Y , Sarver C , Mastellos D , Lambris JD , Cohen GH , Eisenberg RJ , Friedman HM . 2002. Herpes simplex virus type 1 evades the effects of antibody and complement in vivo. J Virol 76:9232–9241. doi:10.1128/jvi.76.18.9232-9241.2002 12186907 PMC136467

[38] Lubinski JM , Lazear HM , Awasthi S , Wang F , Friedman HM . 2011. The herpes simplex virus 1 IgG Fc receptor blocks antibody-mediated complement activation and antibody-dependent cellular cytotoxicity in vivo. J Virol 85:3239–3249. doi:10.1128/JVI.02509-10 21228231 PMC3067879

[39] Para MF , Goldstein L , Spear PG . 1982. Similarities and differences in the Fc-binding glycoprotein (gE) of herpes Simplex virus types 1 and 2 and tentative mapping of the viral gene for this glycoprotein. J Virol 41:137–144. doi:10.1128/jvi.41.1.137-144.1982 6283108 PMC256734

[40] Johnson DC , Frame MC , Ligas MW , Cross AM , Stow ND . 1988. Herpes simplex virus immunoglobulin G Fc receptor activity depends on a complex of two viral glycoproteins, gE and gI. J Virol 62:1347–1354. doi:10.1128/jvi.62.4.1347-1354.1988 2831396 PMC253147

[41] Friedman HM , Cohen GH , Eisenberg RJ , Seidel CA , Cines DB . 1984. Glycoprotein C of herpes simplex virus 1 acts as a receptor for the C3b complement component on infected cells. Nature 309:633–635. doi:10.1038/309633a0 6328323

[42] Fries LF , Friedman HM , Cohen GH , Eisenberg RJ , Hammer CH , Frank MM . 1986. Glycoprotein C of herpes simplex virus 1 is an inhibitor of the complement cascade. J Immunol 137:1636–1641. doi:10.4049/jimmunol.137.5.1636 3018078

[43] McNearney TA , Odell C , Holers VM , Spear PG , Atkinson JP . 1987. Herpes simplex virus glycoproteins gC-1 and gC-2 bind to the third component of complement and provide protection against complement-mediated neutralization of viral infectivity. J Exp Med 166:1525–1535. doi:10.1084/jem.166.5.1525 2824652 PMC2189652

[44] Komala Sari T , Gianopulos KA , Nicola AV . 2020. Glycoprotein C of herpes simplex virus 1 shields glycoprotein B from antibody neutralization. J Virol 94:e01852-19. doi:10.1128/JVI.01852-19 31826995 PMC7022361

[45] Medina RA , Stertz S , Manicassamy B , Zimmermann P , Sun X , Albrecht RA , Uusi-Kerttula H , Zagordi O , Belshe RB , Frey SE , Tumpey TM , García-Sastre A . 2013. Glycosylations in the globular head of the hemagglutinin protein modulate the virulence and antigenic properties of the H1N1 influenza viruses. Sci Transl Med 5:187ra70. doi:10.1126/scitranslmed.3005996 PMC3940933 23720581

[46] Li L , Coelingh KL , Britt WJ . 1995. Human cytomegalovirus neutralizing antibody-resistant phenotype is associated with reduced expression of glycoprotein H. J Virol 69:6047–6053. doi:10.1128/JVI.69.10.6047-6053.1995 7666509 PMC189501

[47] Walsh EE , Falsey AR , Sullender WM . 1998. Monoclonal antibody neutralization escape mutants of respiratory syncytial virus with unique alterations in the attachment (G) protein. J Gen Virol 79 ( Pt 3):479–487. doi:10.1099/0022-1317-79-3-479 9519826

[48] Schønning K , Lund O , Lund OS , Hansen JE . 1999. Stoichiometry of monoclonal antibody neutralization of T-cell line-adapted human immunodeficiency virus type 1. J Virol 73:8364–8370. doi:10.1128/JVI.73.10.8364-8370.1999 10482587 PMC112854

[49] Vzorov AN , Compans RW . 2000. Effect of the cytoplasmic domain of the simian immunodeficiency virus envelope protein on incorporation of heterologous envelope proteins and sensitivity to neutralization. J Virol 74:8219–8225. doi:10.1128/jvi.74.18.8219-8225.2000 10954518 PMC116329

[50] Klasse PJ . 2014. Neutralization of virus infectivity by antibodies: old problems in new perspectives. Adv Biol 2014:157895. doi:10.1155/2014/157895 27099867 PMC4835181

[51] Yuhasz SA , Stevens JG . 1993. Glycoprotein B is a specific determinant of herpes simplex virus type 1 neuroinvasiveness. J Virol 67:5948–5954. doi:10.1128/JVI.67.10.5948-5954.1993 8396662 PMC238015

[52] Imai T , Arii J , Minowa A , Kakimoto A , Koyanagi N , Kato A , Kawaguchi Y . 2011. Role of the herpes simplex virus 1 Us3 kinase phosphorylation site and endocytosis motifs in the intracellular transport and neurovirulence of envelope glycoprotein B. J Virol 85:5003–5015. doi:10.1128/JVI.02314-10 21389132 PMC3126194

[53] Bagdonaite I , Vakhrushev SY , Joshi HJ , Wandall HH . 2018. Viral glycoproteomes: technologies for characterization and outlook for vaccine design. FEBS Lett 592:3898–3920. doi:10.1002/1873-3468.13177 29961944

[54] Luo S , Hu K , He S , Wang P , Zhang M , Huang X , Du T , Zheng C , Liu Y , Hu Q . 2015. Contribution of N-linked glycans on HSV-2 gB to cell-cell fusion and viral entry. Virology 483:72–82. doi:10.1016/j.virol.2015.04.005 25965797

[55] Vallbracht M , Klupp BG , Mettenleiter TC . 2021. Influence of N-glycosylation on expression and function of pseudorabies virus glycoprotein gB. Pathogens 10:61. doi:10.3390/pathogens10010061 33445487 PMC7827564

[56] Maruzuru Y , Ichinohe T , Sato R , Miyake K , Okano T , Suzuki T , Koshiba T , Koyanagi N , Tsuda S , Watanabe M , Arii J , Kato A , Kawaguchi Y . 2018. Herpes simplex virus 1 VP22 inhibits AIM2-dependent Inflammasome activation to enable efficient viral replication. Cell Host Microbe 23:254–265. doi:10.1016/j.chom.2017.12.014 29447697

[57] Kato A , Adachi S , Kawano S , Takeshima K , Watanabe M , Kitazume S , Sato R , Kusano H , Koyanagi N , Maruzuru Y , Arii J , Hatta T , Natsume T , Kawaguchi Y . 2020. Identification of a herpes simplex virus 1 gene encoding neurovirulence factor by chemical proteomics. Nat Commun 11:4894. doi:10.1038/s41467-020-18718-9 32994400 PMC7524712

[58] Tanaka M , Kagawa H , Yamanashi Y , Sata T , Kawaguchi Y . 2003. Construction of an excisable bacterial artificial chromosome containing a full-length infectious clone of herpes simplex virus type 1: viruses reconstituted from the clone exhibit wild-type properties in vitro and in vivo. J Virol 77:1382–1391. doi:10.1128/JVI.77.2.1382-1391.2003 12502854 PMC140785

[59] Hirohata Y , Kato A , Oyama M , Kozuka-Hata H , Koyanagi N , Arii J , Kawaguchi Y . 2015. Interactome analysis of herpes simplex virus 1 envelope glycoprotein H. Microbiol Immunol 59:331–337. doi:10.1111/1348-0421.12255 25808324

[60] Maruzuru Y , Koyanagi N , Kato A , Kawaguchi Y . 2021. Role of the DNA binding activity of herpes simplex virus 1 VP22 in evading Aim2-dependent Inflammasome activation induced by the virus. J Virol 95:e02172-20. doi:10.1128/JVI.02172-20 33298538 PMC8092817

[61] Arii J , Takeshima K , Maruzuru Y , Koyanagi N , Nakayama Y , Kato A , Mori Y , Kawaguchi Y . 2022. Role of the arginine cluster in the disordered domain of herpes simplex virus 1 UL34 for the recruitment of ESCRT-III for viral primary envelopment. J Virol 96:e0170421. doi:10.1128/JVI.01704-21 34730397 PMC8791252

[62] Sugimoto K , Uema M , Sagara H , Tanaka M , Sata T , Hashimoto Y , Kawaguchi Y . 2008. Simultaneous tracking of capsid, tegument, and envelope protein localization in living cells infected with triply fluorescent herpes simplex virus 1. J Virol 82:5198–5211. doi:10.1128/JVI.02681-07 18353954 PMC2395178

[63] Zufferey R , Nagy D , Mandel RJ , Naldini L , Trono D . 1997. Multiply attenuated Lentiviral vector achieves efficient gene delivery in vivo. Nat Biotechnol 15:871–875. doi:10.1038/nbt0997-871 9306402

[64] Kato A , Tanaka M , Yamamoto M , Asai R , Sata T , Nishiyama Y , Kawaguchi Y . 2008. Identification of a physiological phosphorylation site of the herpes simplex virus 1-encoded protein kinase US3 which regulates its optimal catalytic activity in vitro and influences its function in infected cells. J Virol 82:6172–6189. doi:10.1128/JVI.00044-08 18417577 PMC2447112

[65] Kato A , Oda S , Watanabe M , Oyama M , Kozuka-Hata H , Koyanagi N , Maruzuru Y , Arii J , Kawaguchi Y . 2018. Roles of the phosphorylation of herpes simplex virus 1 UL51 at a specific site in viral replication and Pathogenicity. J Virol 92:e01035-18. doi:10.1128/JVI.01035-18 29976672 PMC6146714

[66] Satoh T , Arii J , Suenaga T , Wang J , Kogure A , Uehori J , Arase N , Shiratori I , Tanaka S , Kawaguchi Y , Spear PG , Lanier LL , Arase H . 2008. Pilralpha is a herpes simplex virus-1 entry coreceptor that associates with glycoprotein B. Cell 132:935–944. doi:10.1016/j.cell.2008.01.043 18358807 PMC2394663

[67] Kawaguchi Y , Bruni R , Roizman B . 1997. Interaction of herpes simplex virus 1 alpha regulatory protein ICP0 with elongation factor 1Delta: ICP0 affects translational machinery. J Virol 71:1019–1024. doi:10.1128/jvi.71.2.1019-1024.1997 8995621 PMC191152

[68] Kawaguchi Y , Van Sant C , Roizman B . 1997. Herpes simplex virus 1 alpha regulatory protein ICP0 interacts with and stabilizes the cell cycle regulator cyclin D3. J Virol 71:7328–7336. doi:10.1128/jvi.71.10.7328-7336.1997 9311810 PMC192077

[69] Arii J , Takeshima K , Maruzuru Y , Koyanagi N , Kato A , Kawaguchi Y , Jung JU . 2019. Roles of the Interhexamer contact site for hexagonal lattice formation of the herpes simplex virus 1 nuclear egress complex in viral primary envelopment and replication. J Virol 93:e00498-19. doi:10.1128/JVI.00498-19 31043535 PMC6600192

[70] Tanaka M , Nishiyama Y , Sata T , Kawaguchi Y . 2005. The role of protein kinase activity expressed by the UL13 gene of herpes simplex virus 1: the activity is not essential for optimal expression of UL41 and ICP0. Virology 341:301–312. doi:10.1016/j.virol.2005.07.010 16095647

[71] Sagou K , Imai T , Sagara H , Uema M , Kawaguchi Y . 2009. Regulation of the catalytic activity of herpes simplex virus 1 protein kinase Us3 by autophosphorylation and its role in pathogenesis. J Virol 83:5773–5783. doi:10.1128/JVI.00103-09 19297494 PMC2681960

[72] Park SJ , Lee J , Qi Y , Kern NR , Lee HS , Jo S , Joung I , Joo K , Lee J , Im W . 2019. CHARMM-GUI glycan modeler for modeling and simulation of carbohydrates and glycoconjugates. Glycobiology 29:320–331. doi:10.1093/glycob/cwz003 30689864 PMC6422236

[73] Jo S , Kim T , Iyer VG , Im W . 2008. CHARMM-GUI: a web-based graphical user interface for CHARMM. J Comput Chem 29:1859–1865. doi:10.1002/jcc.20945 18351591

[74] Brooks BR , Brooks CL , Mackerell AD , Nilsson L , Petrella RJ , Roux B , Won Y , Archontis G , Bartels C , Boresch S , Caflisch A , Caves L , Cui Q , Dinner AR , Feig M , Fischer S , Gao J , Hodoscek M , Im W , Kuczera K , Lazaridis T , Ma J , Ovchinnikov V , Paci E , Pastor RW , Post CB , Pu JZ , Schaefer M , Tidor B , Venable RM , Woodcock HL , Wu X , Yang W , York DM , Karplus M . 2009. CHARMM: the Biomolecular simulation program. J Comput Chem 30:1545–1614. doi:10.1002/jcc.21287 19444816 PMC2810661

[75] Winn MD , Ashton AW , Briggs PJ , Ballard CC , Patel P . 2002. Ongoing developments in CCP4 for high-throughput structure determination. Acta Crystallogr D Biol Crystallogr 58:1929–1936. doi:10.1107/s0907444902016116 12393924

[76] Kong L , Sheppard NC , Stewart-Jones GBE , Robson CL , Chen H , Xu X , Krashias G , Bonomelli C , Scanlan CN , Kwong PD , Jeffs SA , Jones IM , Sattentau QJ . 2010. Expression-system-dependent modulation of HIV-1 envelope glycoprotein antigenicity and immunogenicity. J Mol Biol 403:131–147. doi:10.1016/j.jmb.2010.08.033 20800070 PMC2950005

[77] Urbanowicz RA , Wang R , Schiel JE , Keck Z-Y , Kerzic MC , Lau P , Rangarajan S , Garagusi KJ , Tan L , Guest JD , Ball JK , Pierce BG , Mariuzza RA , Foung SKH , Fuerst TR . 2019. Antigenicity and immunogenicity of differentially glycosylated hepatitis C virus E2 envelope proteins expressed in mammalian and insect cells. J Virol 93:e01403-18. doi:10.1128/JVI.01403-18 PMC6430559 30651366

