# Supplementary figures and images for: Dual impacts of a glycan shield on the envelope glycoprotein B of HSV-1: evasion from human antibodies in vivo and neurovirulence
Source: mBio. 2023 Jun 27;14(4):e00992-23. doi: 10.1128/mbio.00992-23 (PMC10470582; doi:10.1128/mbio.00992-23)

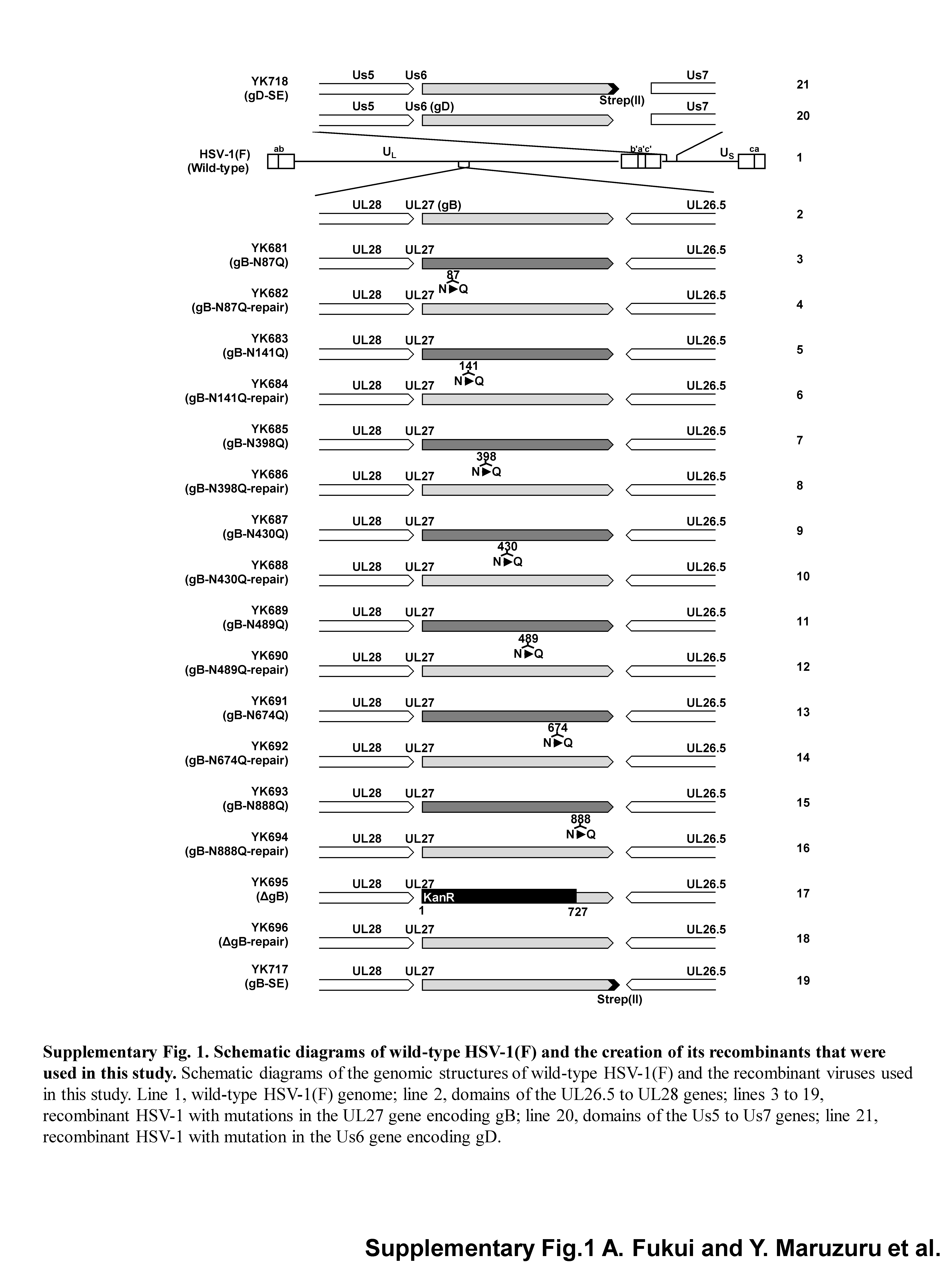

Supplement: Fig. S1 — Schematic diagrams of wild-type HSV-1(F) and the creation of its recombinants that were used in this study. [file mbio.00992-23-s0001.tif]

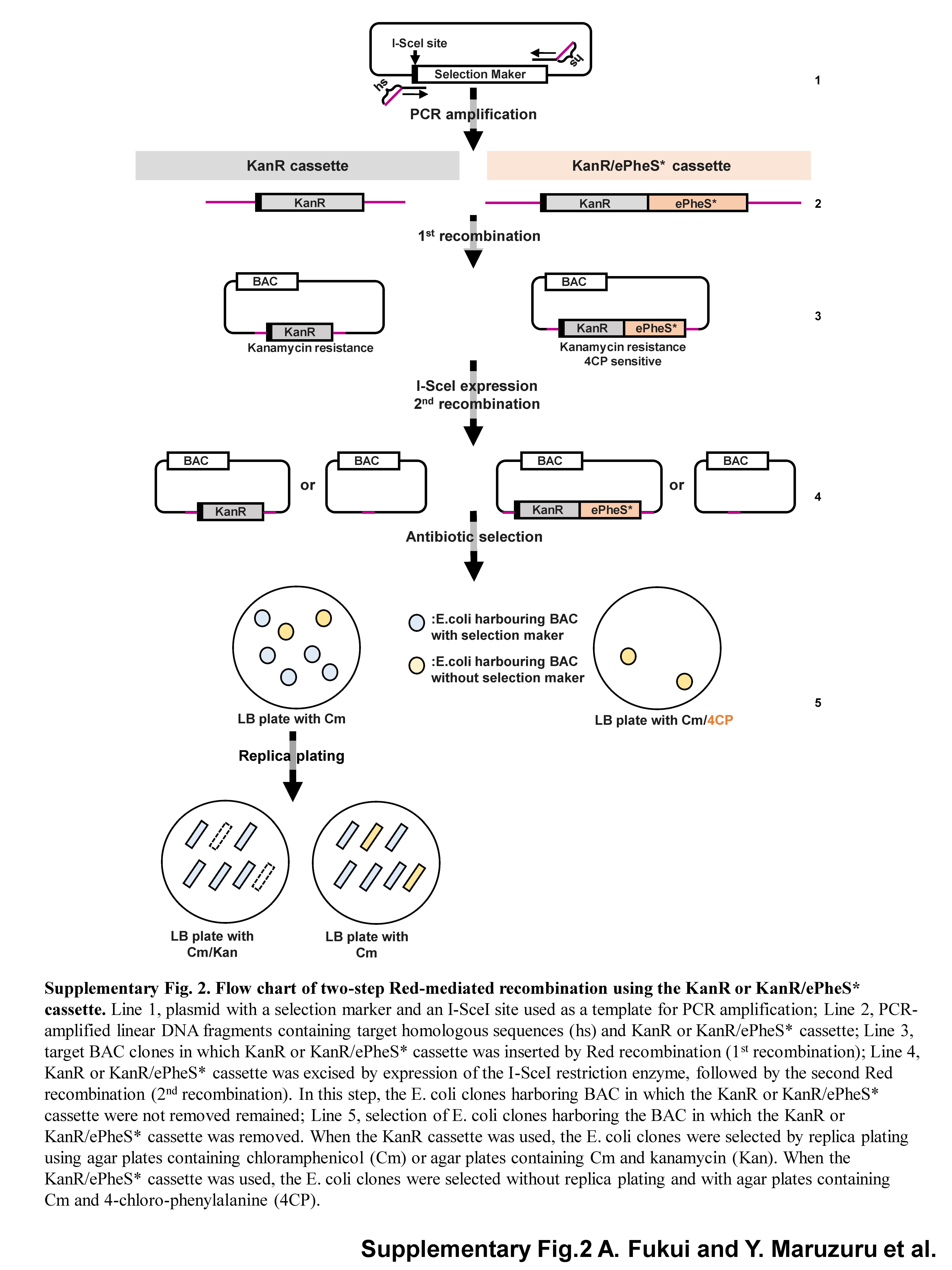

Supplement: Fig. S2 — Flow chart of two-step Red-mediated recombination using the KanR or KanR/ePheS* cassette. [file mbio.00992-23-s0002.tif]

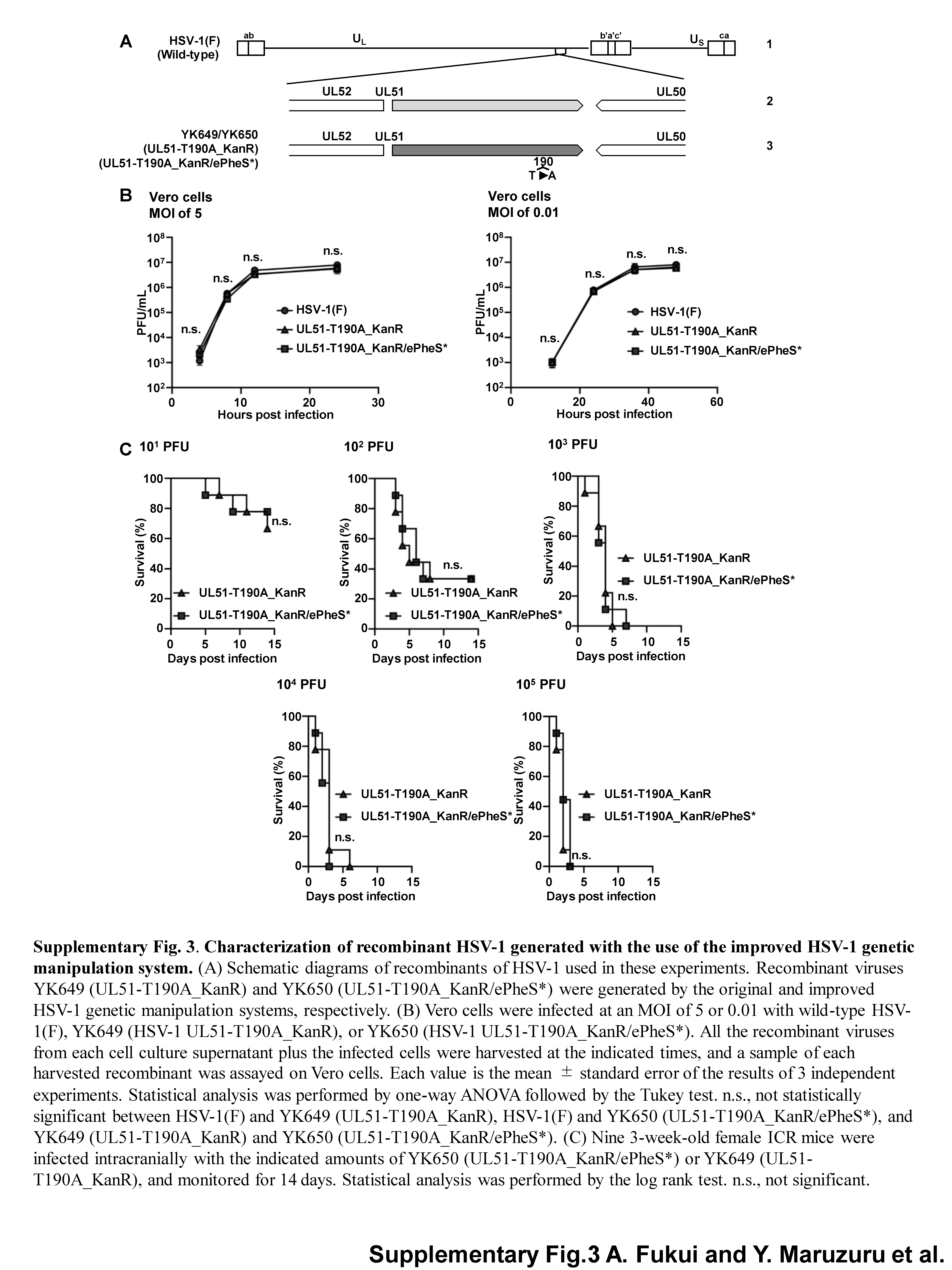

Supplement: Fig. S3 — Characterization of recombinant HSV-1 generated with the use of the improved HSV-1 genetic manipulation system. [file mbio.00992-23-s0003.tif]

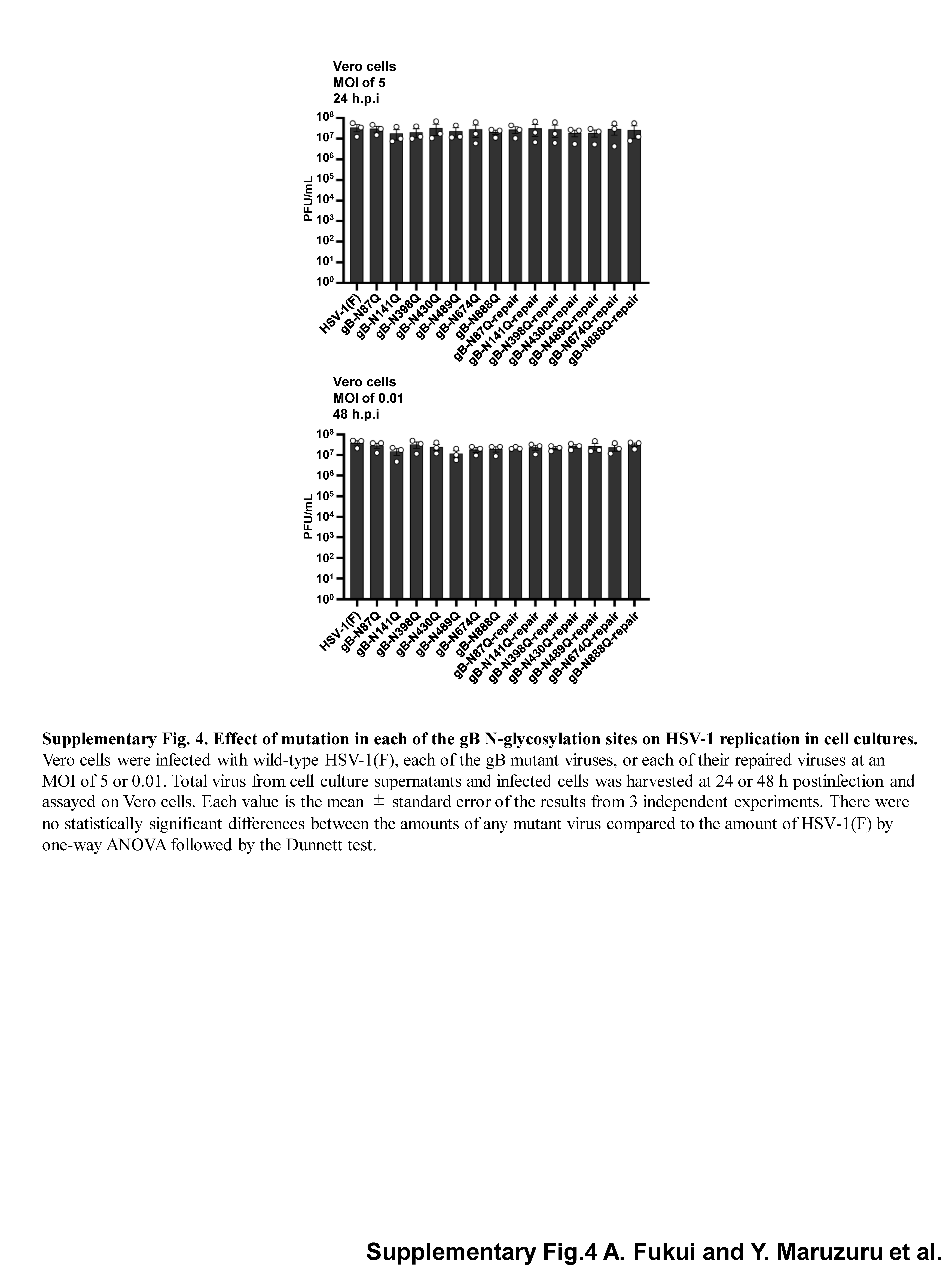

Supplement: Fig. S4 — Effect of mutation in each of the gB N-glycosylation sites on HSV-1 replication in cell cultures. [file mbio.00992-23-s0004.tif]

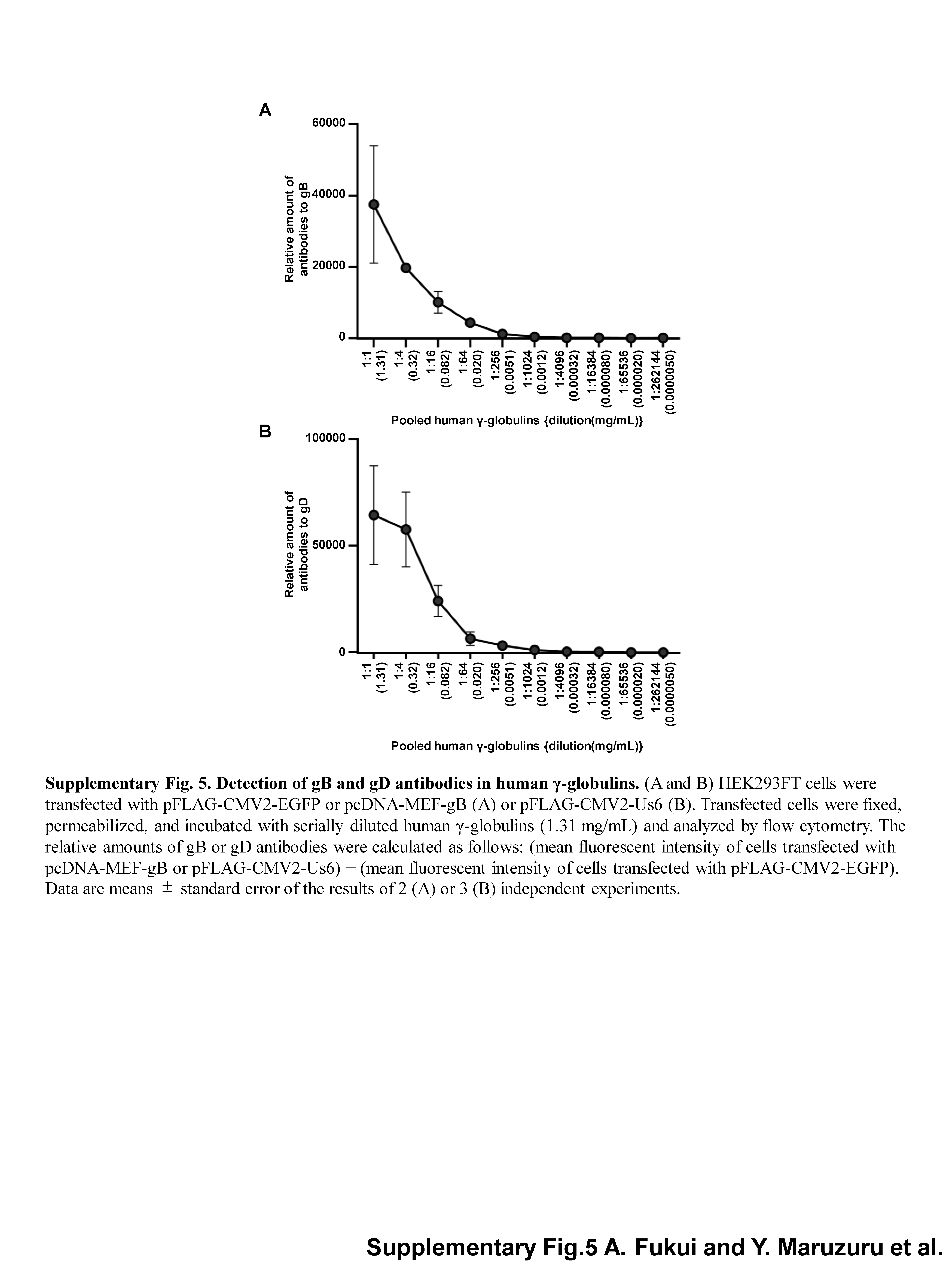

Supplement: Fig. S5 — Detection of gB and gD antibodies in human γ-globulins. [file mbio.00992-23-s0005.tif]

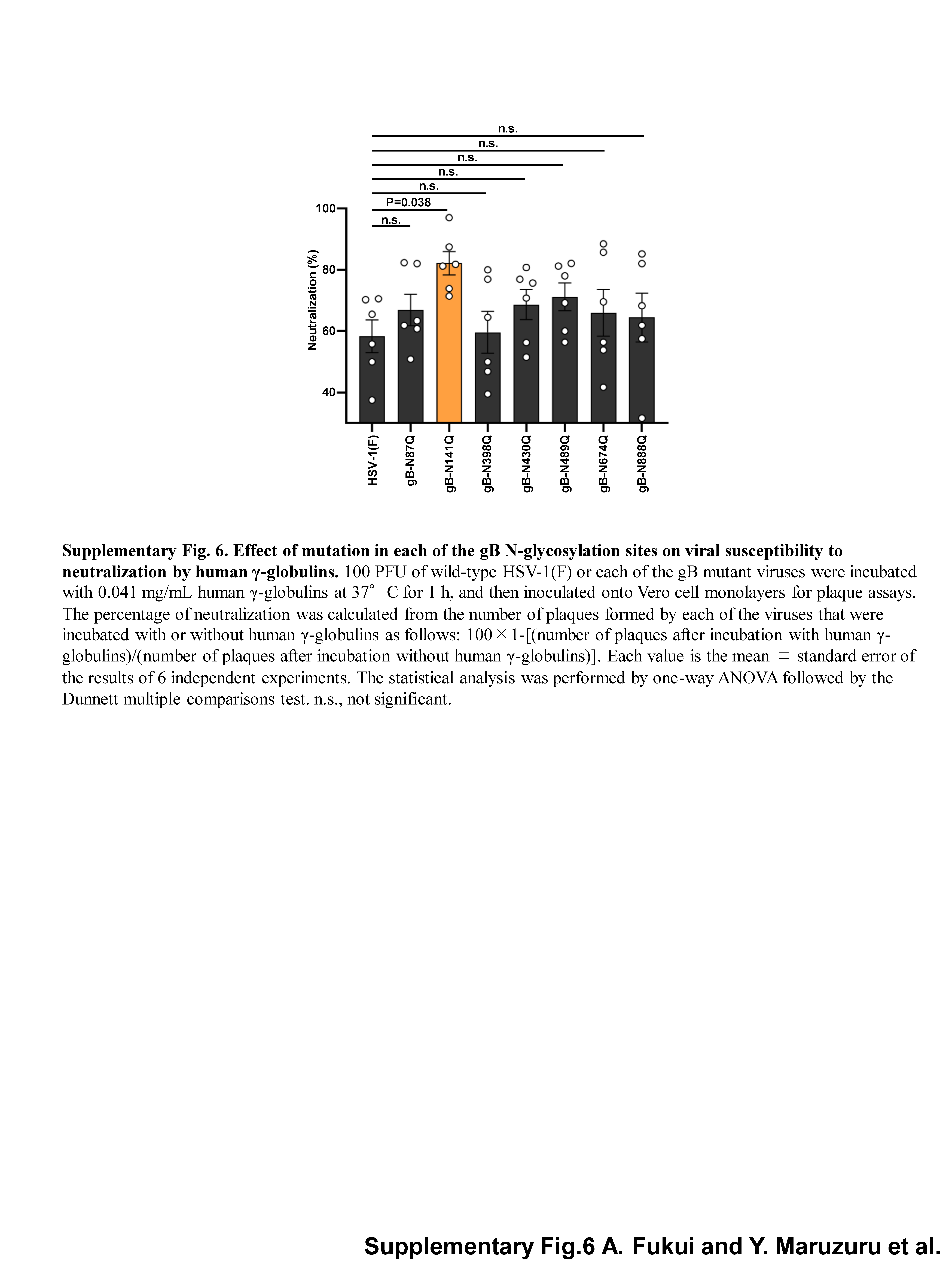

Supplement: Fig. S6 — Effect of mutation in each of the gB N-glycosylation sites on viral susceptibility to neutralization by human γ-globulins. [file mbio.00992-23-s0006.tif]

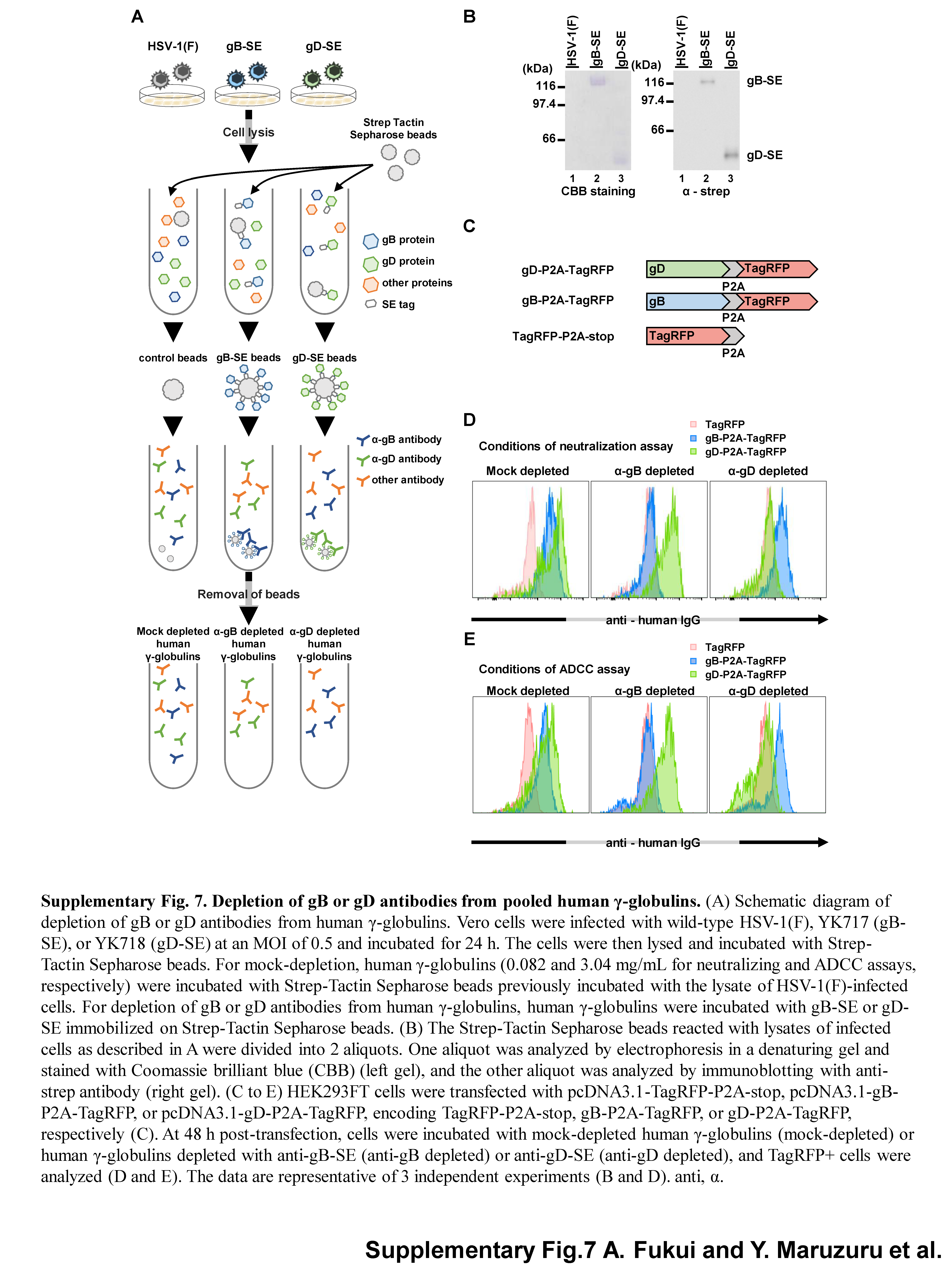

Supplement: Fig. S7 — Depletion of gB or gD antibodies from pooled human γ-globulins. [file mbio.00992-23-s0007.tif]

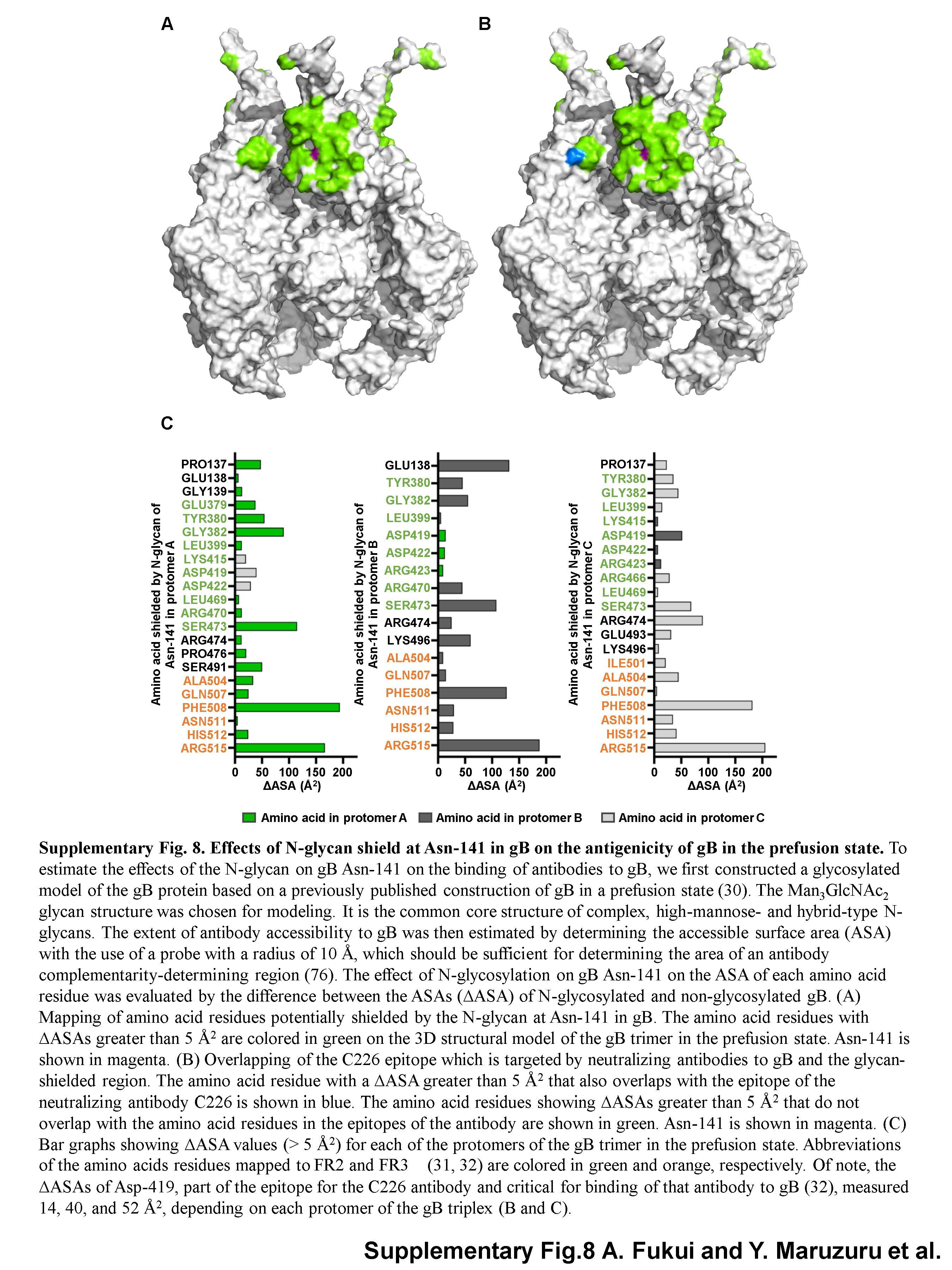

Supplement: Fig. S8 — Effects of N-glycan shield at Asn-141 in gB on the antigenicity of gB in the prefusion state. [file mbio.00992-23-s0008.tif]
