# Supplementary material for: Dual impacts of a glycan shield on the envelope glycoprotein B of HSV-1: evasion from human antibodies in vivo and neurovirulence
Source: mBio. 2023 Jun 27;14(4):e00992-23. doi: 10.1128/mbio.00992-23 (PMC10470582; doi:10.1128/mbio.00992-23)
Supplement: Table S1 — Efficiency of two-step Red-mediated recombination and information on oligonucleotides. [file mbio.00992-23-s0009.docx]

**Supplementary Table 1A. Efficiency of two-step Red-mediated recombination with ePheS* cassette**

| Mutation inserted into pYEbacC102Cre | Selection cassette | Selection cassette insertion^a^ | Selection cassette excision^b^ | |
| --- | --- | --- | --- | --- |
|  |  |  | Selection plate |  |
| Alanine substitution of UL51 T190 | KanR | 18/20 (90%) | Cm | 4/23 (17.4%) |
|  | KanR/ePheS* | 15/20 (75.0%) | **Cm/4CP** | **20/23 (87.0%)** |
| Deletion of UL54, codons 1-513 | KanR | 14/15 (93.3%) | Cm | 4/23 (17.4%) |
|  | KanR/ePheS* | 12/15 (80.0%) | **Cm/4CP** | **18/19 (94.7%)** |
| Insertion of Flag-tag to N-terminus of ICP22 | KanR | 13/15 (86.7%) | Cm | 2/23 (8.7%) |
|  | KanR/ePheS* | 13/15 (86.7%) | **Cm/4CP** | **20/23 (87.0%)** |

^a^As determined by colony PCR. Selection cassette inserted colonies/total colonies

^b^As determined by colony PCR. Colonies without selection cassette as desired/total colonies

**Supplementary Table 1B Oligonucleotide sequences and DNA templates for the construction of plasmids**

| Constructed plasmid | Oligonucleotide sequence (5′-3′) | PCR DNA template | Recipient plasmid |
| --- | --- | --- | --- |
| PBS-KanR-ePheS* | 5'-GCGATATCAGGATGACGACGATAAGTAG-3' | pEP-KanS (23) | pBluescript II KS(+) (Stratagene) |
|  | 5'-TCCCGTTGAATATGGCTCATAACACCCCTTGTATTACTGTTTATG-3' |  |  |
|  | 5'-CATAAACAGTAATACAAGGGGTGTTATGAGCCATATTCAACGGGA-3' | pUC18K ePAG2 (24) |  |
|  | 5'-GCGCGGCCGCTGCAAGCAGCAGATTACGCG-3' |  |  |
| pBS-TEV-2xStrep-KanS | 5'-GCGAATTCGGAGGTTCAGAGAATTTGTATTTTCAGGGTGCTAGCTGGTGTCATCCTCAATTTGAGAAGGGTGGAGGTGCCCGAGGTGGAGGATGACGACGATAAGTAGGG-3' | pEP-KanS (23) | pBluescript II KS(+) (Stratagene) |
|  | 5'-GCGGATCCTTTTTCGAACTGCGGGTGGCTCCACGATCCACCTCCCGATCCACCTCGGGCACCTCCACCCTTCTCAAATTGAGGATGACCAACCAATTAACCAATTCTGATTAG-3' |  |  |
| pcDNA3.1-TagRFP-P2A | 5′-GCGGATCCGCCACCATGGTGTCTAAGGGCGAAGA-3′ | pTagRFP-N1 (61) | pcDNA3.1 (Invitrogen) |
|  | 5′-GCGAATTCAGGCCCGGGGTTTTCTTCAACATCTCCTGCTTGCTTTAACAGAGAGAAGTTCGTGGCTCCGCTTCCATTAAGTTTGTGCCCCAGTTTG-3′ |  |  |
| pcDNA3.1-P2A-TagRFP | 5′-GCGATATCGGAAGCGGAGCCACGAACTTCTCTCTGTTAAAGCAAGCAGGAGATGTTGAAGAAAACCCCGGGCCTGTGTCTAAGGGCGAAGAGCT-3′ | pTagRFP-N1 (61) | pcDNA3.1 (Invitrogen) |
|  | 5′-GCGCGGCCGCTCAATTAAGTTTGTGCCCCAGTT-3′ |  |  |
| pcDNA3.1-TagRFP-P2A-stop | 5′-CCCCGGGCCTGAATTCTGAGATATCCAGCACAGTGGCGG-3′ | N/A | pcDNA3.1-TagRFP-P2A (This study) |
|  | 5′-CCGCCACTGTGCTGGATATCTCAGAATTCAGGCCCGGGG-3′ |  |  |
| pcDNA3.1-gB-P2A-TagRFP | 5′-CCAGTGTGGTGGAATTCGCCACCATGCGCCAGGGCGCCCCCGC-3′ | HSV-1(F) genome | pcDNA3.1-P2A-TagRFP (This study) |
|  | 5′-GGCTCCGCTTCCGATCAGGTCGTCCTCGTCGGCGT-3′ |  |  |
| pcDNA3.1-gD-P2A-TagRFP | 5′-CCAGTGTGGTGGAATTCGCCACCATGGGGGGGGCTGCCGCCAG-3′ | HSV-1(F) genome | pcDNA3.1-P2A-TagRFP (This study) |
|  | 5′-GGCTCCGCTTCCGATGTAAAACAAGGGCTGGTGCG-3′ |  |  |
| pFLAG-CMV2-Us6 | 5′- GCGAATTCGGGGGGGGCTGCCGCCAGGT-3′ | pYEbac102 (58) | pFLAG-CMV2(Sigma-Aldrich) |
|  | 5′- GCGGATCCCTAGTAAAACAAGGGCTGGT-3′ |  |  |
| pMAL-VP5o-P3 | 5′- GCGAATTCACATTCGTGCTGCCCCTGGG-3′ | pEGFP-VP5o (Genscript) | pMAL-c (New England BioLads) |
|  | 5′- GCGTCGACTCATCCCAGCTCCAGGCCTCTGG-3′ |  |  |

**Supplementary Table 1C Summary of synthesized plasmids**

| Constructed plasmid | Synthesized DNA sequence |
| --- | --- |
| pEGFP-VP5o | ATGGCAGCACCAGCCAGGGATCCACCTGGATATAGATACGCAGCAGCAATGGTGCCAACCGGATCTATCCTGAGCACAATCGAGGTGGCCAGCCACCGGAGACTGTTCGATTTCTTTGCCAGGGTGCGCTCTGACGAGAACAGCCTGTATGACGTGGAGTTTGATGCCCTGCTGGGCAGCTACTGTAATACCCTGTCCCTGGTGCGGTTCCTGGAGCTGGGACTGTCCGTGGCATGCGTGTGCACAAAGTTTCCTGAGCTGGCCTATATGAACGAGGGAAGGGTGCAGTTCGAGGTGCACCAGCCACTGATCGCCAGAGATGGACCACACCCTGTGGAGCAGCCTGTGCACAACTACATGACCAAAGTGATCGACAGGCGCGCCCTGAATGCCGCCTTTTCTCTGGCAACCGAGGCAATCGCCCTGCTGACAGGAGAGGCCCTGGACGGCACCGGAATCAGCCTGCACAGGCAGCTGAGGGCAATCCAGCAGCTGGCAAGGAACGTGCAGGCCGTGCTGGGAGCCTTCGAGAGAGGAACCGCCGATCAGATGCTGCACGTGCTGCTGGAGAAGGCACCACCACTGGCCCTGCTGCTGCCCATGCAGCGGTACCTGGACAATGGCAGACTGGCAACCCGGGTGGCCAGAGCCACACTGGTGGCAGAGCTGAAGCGGTCCTTTTGCGATACCTCTTTCTTTCTGGGCAAGGCAGGACACAGGAGAGAGGCAATCGAGGCCTGGCTGGTGGACCTGACCACAGCCACACAGCCAAGCGTGGCAGTGCCAAGGCTGACCCACGCAGATACACGGGGCAGACCTGTGGACGGCGTGCTGGTGACCACAGCAGCCATCAAGCAGAGACTGCTGCAGTCCTTCCTGAAGGTGGAGGACACAGAGGCCGATGTGCCTGTGACCTACGGCGAGATGGTGCTGAACGGCGCCAATCTGGTGACCGCCCTGGTCATGGGCAAGGCCGTGCGGTCCCTGGACGATGTGGGCAGACACCTGCTGGATATGCAGGAGGAGCAGCTGGAGGCCAACAGAGAGACACTGGACGAGCTGGAGTCTGCCCCACAGACCACAAGGGTGCGCGCAGATCTGGTGGCAATCGGCGACAGGCTGGTGTTTCTGGAGGCCCTGGAGAAGCGCATCTATGCCGCCACCAACGTGCCATACCCCCTGGTGGGCGCCATGGATCTGACATTCGTGCTGCCCCTGGGCCTGTTTAATCCTGCCATGGAGCGGTTCGCAGCACACGCAGGCGACCTGGTGCCTGCCCCAGGCCACCCCGAGCCTAGGGCCTTCCCTCCACGCCAGCTGTTCTTTTGGGGCAAGGACCACCAGGTGCTGAGACTGTCTATGGAGAACGCAGTGGGAACCGTGTGCCACCCCAGCCTGATGAATATCGATGCAGCAGTGGGAGGAGTGAACCACGACCCAGTGGAGGCCGCCAATCCCTATGGCGCCTACGTGGCAGCACCAGCAGGACCTGGAGCAGATATGCAGCAGAGATTCCTGAATGCCTGGCGGCAGAGACTGGCACACGGAAGGGTGCGCTGGGTGGCAGAGTGCCAGATGACCGCAGAGCAGTTTATGCAGCCTGACAACGCCAATCTGGCCCTGGAGCTGCACCCAGCCTTCGATTTCTTTGCAGGAGTGGCAGACGTGGAGCTGCCAGGAGGAGAGGTGCCACCTGCAGGACCAGGAGCCATCCAGGCCACCTGGAGGGTGGTGAACGGCAATCTGCCTCTGGCCCTGTGCCCAGTGGCCTTCAGGGATGCCAGAGGCCTGGAGCTGGGAGTGGGCCGCCACGCAATGGCACCTGCAACAATCGCAGCCGTGAGGGGAGCCTTTGAGGACCGCTCCTATCCAGCCGTGTTCTACCTGCTGCAGGCCGCCATCCACGGCTCTGAGCACGTGTTTTGCGCACTGGCAAGGCTGGTGACCCAGTGTATCACAAGCTATTGGAACAATACCAGATGTGCCGCCTTCGTGAACGATTACTCCCTGGTGTCTTATATCGTGACATACCTGGGAGGCGATCTGCCAGAGGAGTGCATGGCCGTGTACAGGGACCTGGTGGCACACGTGGAGGCCCTGGCCCAGCTGGTGGACGACTTCACCCTGCCAGGACCAGAGCTGGGAGGACAGGCACAGGCAGAGCTGAATCACCTGATGAGGGACCCCGCCCTGCTGCCACCACTGGTGTGGGACTGCGATGGACTGATGAGGCACGCCGCCCTGGACAGGCACAGAGATTGTCGCATCGACGCAGGAGGACACGAGCCCGTGTACGCAGCCGCCTGCAACGTGGCCACCGCCGATTTCAACCGGAATGACGGCAGACTGCTGCACAATACACAGGCCAGGGCCGCAGATGCAGCAGACGATAGGCCACACAGACCAGCAGACTGGACCGTGCACCACAAGATCTACTATTACGTGCTGGTGCCCGCCTTTTCTAGGGGCCGCTGCTGTACCGCAGGCGTGAGGTTCGATCGCGTGTACGCCACACTGCAGAACATGGTGGTGCCTGAGATCGCCCCAGGCGAGGAGTGTCCTTCCGATCCAGTGACCGACCCAGCACACCCTCTGCACCCAGCCAACCTGGTGGCCAACACAGTGAATGCCATGTTTCACAATGGAAGGGTGGTGGTGGACGGACCAGCAATGCTGACCCTGCAGGTGCTGGCCCACAACATGGCCGAGCGCACCACAGCCCTGCTGTGCAGCGCCGCACCAGATGCAGGAGCCAACACCGCCTCCACAGCCAATATGCGCATCTTCGACGGCGCCCTGCACGCAGGCGTGCTGCTGATGGCCCCCCAGCACCTGGACCACACAATCCAGAATGGCGAGTACTTCTACGTGCTGCCTGTGCACGCACTGTTCGCAGGAGCAGATCACGTGGCAAACGCACCCAATTTTCCTCCAGCCCTGAGGGACCTGGCAAGGCACGTGCCTCTGGTGCCACCTGCCCTGGGCGCCAACTATTTCAGCTCCATCAGGCAGCCCGTGGTGCAGCACGCCCGCGAGAGCGCCGCAGGCGAGAATGCCCTGACCTATGCCCTGATGGCCGGCTACTTCAAGATGTCCCCAGTGGCCCTGTACCACCAGCTGAAGACAGGCCTGCACCCCGGCTTCGGCTTTACCGTGGTGCGGCAGGATAGATTTGTGACAGAGAACGTGCTGTTCTCCGAGAGGGCCTCTGAGGCCTATTTTCTGGGCCAGCTGCAGGTGGCAAGGCACGAGACCGGAGGAGGCGTGAACTTCACCCTGACACAGCCTAGGGGCAATGTGGACCTGGGAGTGGGATACACCGCAGTGGCAGCCACCGCCACAGTGCGGAACCCCGTGACAGATATGGGCAACCTGCCTCAGAACTTCTACCTGGGAAGAGGAGCACCACCACTGCTGGACAATGCAGCAGCCGTGTACCTGAGGAACGCAGTGGTGGCAGGAAATAGACTGGGACCTGCACAGCCTCTGCCAGTGTTCGGATGTGCACAGGTGCCAAGGAGGGCAGGAATGGATCACGGACAGGACGCCGTGTGCGAGTTTATCGCCACCCCTGTGGCCACAGACATCAACTATTTCCGGAGACCCTGTAATCCTCGGGGCAGAGCAGCAGGAGGCGTGTATGCAGGCGATAAGGAGGGCGACGTGATCGCCCTGATGTACGATCACGGACAGAGCGACCCAGCAAGGCCCTTTGCCGCAACCGCAAACCCATGGGCAAGCCAGCGGTTCAGCTACGGCGATCTGCTGTATAACGGCGCCTACCACCTGAATGGCGCCTCTCCCGTGCTGAGCCCTTGTTTTAAGTTCTTTACCGCCGCCGACATCACAGCCAAGCACCGGTGCCTGGAGAGACTGATCGTGGAGACCGGAAGCGCCGTGTCCACCGCAACAGCAGCCTCCGATGTGCAGTTTAAGCGGCCTCCAGGCTGCAGAGAGCTGGTGGAGGACCCATGTGGCCTGTTCCAGGAGGCCTACCCTATCACATGCGCCTCCGATCCAGCCCTGCTGAGGTCTGCCCGCGACGGAGAGGCACACGCCAGAGAGACCCACTTCACACAGTATCTGATCTACGACGCCTCTCCCCTGAAGGGCCTGAGCCTG |
| pRetroX-TRE3G-gBo | GGATCCGCCACCATGAGACAGGGAGCACCAGCAAGGGGATGCAGATGGTTCGTGGTGTGGGCACTGCTGGGACTGACACTGGGCGTGCTGGTGGCCAGCGCCGCCCCAAGCTCCCCCGGCACCCCTGGCGTGGCCGCCGCCACACAGGCCGCCAACGGCGGCCCAGCCACCCCAGCACCACCTGCACCAGGACCTGCACCAACCGGCGACACAAAGCCTAAGAAGAACAAGAAGCCAAAGAATCCACCACCTCCAAGGCCAGCAGGCGATAATGCAACCGTGGCAGCAGGACACGCCACACTGAGGGAGCACCTGCGCGATATCAAGGCCGAGAACACCGACGCCAATTTCTACGTGTGCCCACCTCCAACCGGAGCAACAGTGGTGCAGTTTGAGCAGCCACGGAGATGTCCTACCCGCCCAGAGGGCCAGAACTACACAGAGGGCATCGCCGTGGTGTTCAAGGAGAATATCGCCCCCTATAAGTTTAAGGCCACCATGTACTATAAGGACGTGACAGTGTCTCAAGTGTGGTTCGGCCACCGGTACAGCCAGTTCATGGGCATCTTTGAGGACAGAGCCCCCGTGCCTTTTGAGGAAGTGATCGATAAGATCAACGCAAAGGGCGTGTGCCGCAGCACCGCCAAGTATGTGCGGAACAATCTGGAGACCACAGCCTTCCACCGGGACGATCACGAGACAGACATGGAGCTGAAGCCTGCAAATGCAGCAACCAGGACATCCAGGGGATGGCACACCACAGATCTGAAGTACAACCCATCTCGCGTGGAGGCCTTCCACCGGTATGGCACCACAGTGAATTGTATCGTGGAGGAGGTGGATGCCAGAAGCGTGTACCCATATGACGAGTTTGTGCTGGCCACCGGCGATTTCGTGTACATGTCCCCCTTTTACGGCTATCGGGAGGGCTCCCACACCGAGCACACATCTTACGCCGCCGACAGATTCAAGCAGGTGGATGGCTTTTATGCCAGAGACCTGACCACAAAGGCAAGGGCAACCGCACCTACCACAAGGAACCTGCTGACCACACCAAAGTTCACAGTGGCATGGGACTGGGTGCCAAAGAGGCCTTCCGTGTGCACCATGACAAAGTGGCAGGAGGTGGACGAGATGCTGCGGAGCGAGTACGGCGGCTCCTTCAGATTTTCTAGCGATGCCATCAGCACCACCTTCACCACAAACCTGACCGAGTATCCCCTGTCCAGAGTGGATCTGGGCGACTGTATCGGCAAGGATGCCAGAGACGCCATGGATAGGATCTTCGCCAGGCGCTACAATGCCACCCACATCAAGGTCGGCCAGCCCCAGTACTATCTGGCCAACGGCGGCTTTCTGATCGCCTACCAGCCTCTGCTGTCCAATACCCTGGCCGAGCTGTATGTGCGGGAGCACCTGAGAGAGCAGTCTAGGAAGCCCCCTAACCCTACACCACCACCTCCAGGAGCAAGCGCCAATGCATCCGTGGAGAGGATCAAGACCACATCCTCTATCGAGTTCGCCCGCCTGCAGTTTACCTATAACCACATCCAGAGGCACGTGAATGACATGCTGGGAAGGGTGGCAATCGCATGGTGCGAGCTGCAGAACCACGAGCTGACCCTGTGGAATGAGGCCAGGAAGCTGAACCCTAATGCAATCGCAAGCGCCACAGTGGGCCGGAGAGTGTCCGCCAGGATGCTGGGCGACGTGATGGCCGTGTCTACCTGCGTGCCAGTGGCAGCCGATAACGTGATCGTGCAGAATAGCATGAGGATCAGCTCCAGGCCAGGAGCATGTTACTCTAGACCCCTGGTGAGCTTCAGGTACGAGGACCAGGGACCACTGGTGGAGGGACAGCTGGGCGAGAACAATGAGCTGCGGCTGACCAGAGATGCCATCGAGCCTTGTACAGTGGGCCACAGGCGCTACTTCACCTTTGGCGGCGGCTACGTGTATTTTGAGGAGTACGCCTATTCTCACCAGCTGAGCAGGGCCGACATCACCACAGTGTCCACCTTCATCGACCTGAACATCACAATGCTGGAGGATCACGAGTTTGTGCCTCTGGAGGTGTACACCCGGCACGAGATCAAGGACTCTGGCCTGCTGGATTATACAGAGGTGCAGCGGAGAAACCAGCTGCACGACCTGAGATTCGCCGACATCGATACCGTGATCCACGCCGATGCCAATGCAGCAATGTTTGCAGGACTGGGAGCCTTCTTTGAGGGAATGGGCGATCTGGGAAGGGCAGTGGGCAAGGTGGTCATGGGAATCGTGGGAGGAGTGGTGTCCGCCGTGTCTGGCGTGTCTAGCTTCATGAGCAACCCCTTTGGCGCCCTGGCCGTGGGACTGCTGGTGCTGGCAGGACTGGCAGCCGCCTTCTTTGCCTTCAGATACGTGATGAGGCTGCAGTCTAATCCCATGAAGGCCCTGTATCCTCTGACCACAAAGGAGCTGAAGAACCCAACCAATCCAGACGCAAGCGGAGAGGGAGAGGAGGGAGGCGACTTTGATGAGGCAAAGCTGGCAGAGGCAAGGGAGATGATCCGGTACATGGCCCTGGTGTCCGCCATGGAGAGGACAGAGCACAAGGCCAAGAAGAAGGGCACCTCCGCCCTGCTGTCTGCCAAGGTGACAGATATGGTCATGCGCAAGAGGCGCAACACCAATTATACACAGGTGCCCAACAAGGACGGCGATGCCGACGAGGACGATCTGTGAGAATTC |
| pRetroX-TRE3G-ICP4o | GGATCCGCCACCATGGCCAGCGAGAACAAGCAGAGGCCTGGCTCCCCTGGACCAACCGATGGACCACCTCCAACACCATCCCCTGACAGGGATGAGAGAGGCGCCCTGGGATGGGGAGCAGAGACCGAGGAGGGAGGCGACGATCCAGACCACGATCCAGACCACCCCCACGATCTGGACGATGCAAGGAGAGACGGAAGGGCACCAGCAGCAGGCACAGACGCCGGCGAGGATGCCGGCGACGCCGTGTCCCCCCGGCAGCTGGCCCTGCTGGCCTCTATGGTGGAGGAGGCCGTGAGAACCATCCCAACACCCGATCCTGCAGCATCCCCACCTAGGACACCAGCCTTCCGGGCAGACGATGACGATGGCGACGAGTACGACGATGCCGCCGATGCAGCAGGCGACAGGGCACCAGCAAGGGGACGGGCCAGAGAGGCCCCCCTGAGAGGCGCCTATCCAGATCCCACCGACAGACTGAGCCCAAGGCCACCAGCACAGCCTCCAAGGCGCCGGAGACACGGCAGGCGCCGGCCTTCTGCCAGCTCCACATCTAGCGATAGCGGCTCCTCTAGCTCCTCTAGCGCCAGCTCCAGCTCCTCTAGCTCCGATGAAGACGAGGACGATGACGGCAATGATGCCGCCGACAGGGCAAGGGAGGCAAGGGCAGTGGGAAGGGGCCCCTCTAGCGCCGCCCCTGAGGCCCCAGGCCGGACCCCCCCTCCACCCGGCCCTCCACCCCTGAGCGAGGCAGCACCTAAGCCAAGAGCAGCAGCCAGGACACCAGCTGCCTCCGCCGGCCGCATCGAGAGAAGGCGCGCAAGGGCAGCAGTGGCAGGAAGAGACGCAACCGGCAGGTTCACAGCAGGACAGCCAAGGAGAGTGGAGCTGGATGCAGACGCAGCATCCGGAGCCTTTTACGCACGGTATAGAGATGGCTACGTGTCTGGCGAGCCATGGCCTGGAGCAGGACCTCCACCACCTGGCCGGGTGCTGTATGGCGGCCTGGGCGACTCCAGACCTGGCCTGTGGGGCGCCCCAGAGGCCGAGGAGGCCAGGCGCCGGTTCGAGGCATCTGGAGCACCAGCAGCCGTGTGGGCCCCTGAGCTGGGCGATGCAGCACAGCAGTACGCACTGATCACCAGGCTGCTGTATACACCAGACGCAGAGGCAATGGGATGGCTGCAGAACCCCAGAGTGGTGCCTGGCGATGTGGCCCTGGACCAGGCATGCTTCAGAATCAGCGGAGCAGCACGGAACAGCTCTAGCTTTATCACCGGCTCCGTGGCCAGGGCCGTGCCCCACCTGGGCTACGCCATGGCCGCCGGCCGCTTCGGATGGGGACTGGCACACGCAGCAGCAGCAGTGGCAATGTCTAGAAGGTACGACAGAGCCCAGAAGGGCTTTCTGCTGACCAGCCTGAGGAGGGCATATGCACCTCTGCTGGCAAGAGAGAACGCCGCCCTGACAGGAGCAGCAGGCAGCCCAGGAGCAGGAGCAGATGACGAGGGCGTGGCCGCCGCCGTGGTGGCTGCCGCCGCCGCCCCAGGCGAGAGGGCCGTGCCCGCCGGCTATGGCGCCGCCGGCATCCTGGCCGCCCTGGGCCGCCTGAGCGCCGCCCCCGCCTCCCCTGCCGGCGGCGATGACCCCGATGCCGCCCGGCACGCCGACGCCGATGACGATGCAGGCAGAAGGGCACAGGCAGGAAGGGTGGCCGTGGAGTGCCTGGCCGCCTGTCGCGGCATCCTGGAGGCCCTGGCCGAGGGCTTCGATGGCGACCTGGCAGCAGTGCCAGGACTGGCAGGAGCACGCCCCGCCTCTCCACCCCGGCCAGAGGGCCCCGCCGGCCCTGCCAGCCCTCCACCCCCTCACGCCGACGCCCCCAGGCTGCGCGCCTGGCTGAGGGAGCTGCGCTTTGTGCGGGATGCCCTGGTGCTGATGCGGCTGAGAGGCGACCTGCGGGTGGCAGGAGGCTCTGAGGCAGCAGTGGCAGCCGTGAGAGCCGTGAGCCTGGTGGCCGGCGCCCTGGGCCCAGCCCTGCCCAGAGATCCTAGGCTGCCATCCTCTGCCGCAGCAGCAGCAGCAGACCTGCTGTTCGAGAACCAGAGCCTGCGGCCTCTGCTGGCCGCCGCCGCCTCCGCCCCAGATGCAGCAGACGCACTGGCAGCAGCAGCAGCATCCGCCGCCCCCAGAGAGGGAAGGAAGAGGAAGTCTCCAGGACCAGCAAGACCACCAGGCGGAGGAGGACCTAGGCCTCCAAAGACCAAGAAGTCCGGCGCCGATGCCCCAGGCTCTGACGCCAGGGCCCCTCTGCCAGCCCCCCCTTCCACACCACCCGGCCCCGAGCCTGCCCCAGCACAGCCAGCAGCACCTAGGGCAGCAGCAGCCCAGGCCCGGCCCAGACCTGTGGCCCTGAGCCGCCGGCCTGCCGAGGGCCCAGACCCCCTGGGCGGCTGGAGAAGGCAGCCTCCAGGCCCTTCCCACACCGCAGCACCAGCAGCAGCCGCCCTGGAGGCCTACTGCTCTCCAAGAGCCGTGGCCGAGCTGACAGATCACCCACTGTTCCCTGTGCCATGGAGGCCCGCCCTGATGTTTGACCCTCGGGCCCTGGCCTCCATCGCCGCCAGATGCGCAGGACCAGCAGCAGCAGCACAGGCAGCCTGTGGCGGCGGCGACGATGACGATAATCCACACCCTCACGGAGCAGCAGGAGGCAGACTGTTTGGCCCACTGAGGGCATCTGGACCACTGAGGAGGATGGCAGCCTGGATGAGGCAGATCCCAGATCCCGAGGACGTGCGCGTGGTGGTGCTGTATAGCCCTCTGCCAGGCGAGGACCTGGCAGGAGGAGGAGCAAGCGGAGGACCACCTGAGTGGTCCGCCGAGAGGGGAGGACTGTCTTGCCTGCTGGCCGCCCTGGCAAACAGGCTGTGCGGCCCCGATACCGCAGCATGGGCAGGAAATTGGACAGGAGCACCTGACGTGAGCGCCCTGGGAGCACAGGGCGTGCTGCTGCTGTCCACCAGAGATCTGGCCTTCGCCGGAGCAGTGGAGTTTCTGGGACTGCTGGCAAGCGCCGGCGACAGAAGGCTGATCGTGGTGAACACAGTGCGGGCATGCGATTGGCCAGCAGACGGACCCGCCGTGTCCAGACAGCACGCATACCTGGCATGCGATCTGCTGCCAGCAGTGCAGTGTGCCGTGCGCTGGCCTGCAGCAAGGGACCTGAGGCGGACCGTGCTGGCCCCTGGCAGGGTGTTCGGCCCAGGCGTGTTTGCAAGAGTGGAGGCAGCACACGCAAGGCTGTACCCCGACGCACCACCACTGAGGCTGTGCAGGGGAGGCAATGTGAGGTATCGCGTGCGGACCAGATTCGGCCCTGATACACCCGTGCCTATGTCTCCAAGGGAGTACAGAAGGGCCGTGCTGCCTGCCCTGGACGGAAGGGCAGCAGCAAGCGGAACCACAGATGCAATGGCACCAGGAGCACCAGACTTTTGCGAGGAGGAGGCACACAGCCACAGGGCATGTGCAAGATGGGGACTGGGAGCACCTCTGAGGCCAGTGTATGTGGCCCTGGGCAGAGAGGCCGTGAGGGCAGGACCAGCAAGGTGGAGGGGACCTAGGAGGGACTTCTGTGCAAGGGCCCTGCTGGAGCCAGACGATGACGCACCTCCACTGGTGCTGAGGGGAGGCGATGACGATGACGATGGACCAGGCGCCCTGCCACCTGCCCTGCCTGGCATCCGCTGGGCCTCTGCCACCGGCCGGAGCGGCACAGTGCTGGCCGCCGCCGGCGCCGTGGAGGTGCTGGGCGCCGAGGCCGGCCTGGCCACCCCACCCAGAAGGGAGGTGGTGGATTGGGAGGGAGCATGGGACGATGACGATGGAGGAGCCTTTGAGGGCGACGGCGTGCTGTGAGAATTC |

**Supplementary Table 1D Oligonucleotide sequences, plasmid, and E. coli GS1873 containing HSV-BAC for the construction of recombinant viruses or mutagenesis.**

| Recombinant virus | Mutation inserted into pYEbac102Cre | Oligonucleotide sequence (5′-3′) | Plasmid DNA template | *E*. *coli* GS1873 containing HSV-BAC |
| --- | --- | --- | --- | --- |
| YK681 | HSV-1 gB-N87Q | 5′-AAAACCGAAAAACCCACCGCCGCCGCGCCCCGCCGGCGACCAGGCGACCGTCGCCGCGGGAGGATGACGACGATAAGTAG-3′ | pBS-KanR-ePheS* (This study) | *E. coli* GS1783/pYEbac102Cre (65) |
|  |  | 5′-GCTCGCGCAGGGTGGCGTGGCCCGCGGCGACGGTCGCCTGGTCGCCGGCGGGGCGCGGCGTGCAAGCAGCAGATTACGCG-3′ |  |  |
| YK683 | HSV-1 gB-N141Q | 5′-CGAGCAGCCGCGCCGCTGCCCGACCCGGCCCGAGGGTCAGCAGTACACGGAGGGCATCGCAGGATGACGACGATAAGTAG-3′ | pBS-KanR-ePheS* (This study) | *E. coli* GS1783/pYEbac102Cre (65) |
|  |  | 5′-TGTTCTCCTTGAAGACCACCGCGATGCCCTCCGTGTACTGCTGACCCTCGGGCCGGGTCGTGCAAGCAGCAGATTACGCG-3′ |  |  |
| YK685 | HSV-1 gB-N398Q | 5′-CCGATTCTCCTCCGACGCCATATCCACCACCTTCACCACCCAGCTGACCGAGTACCCGCTAGGATGACGACGATAAGTAG-3′ | pBS-KanR-ePheS* (This study) | *E. coli* GS1783/pYEbac102Cre (65) |
|  |  | 5′-CCCCCAGGTCCACGCGCGAGAGCGGGTACTCGGTCAGCTGGGTGGTGAAGGTGGTGGATATGCAAGCAGCAGATTACGCG-3′ |  |  |
| YK687 | HSV-1 gB-N430Q | 5′-CGCCCGCGACGCCATGGACCGCATCTTCGCCCGCAGGTACCAGGCGACGCACATCAAGGTAGGATGACGACGATAAGTAG-3′ | pBS-KanR-ePheS* (This study) | *E. coli* GS1783/pYEbac102Cre (65) |
|  |  | 5′-GGTAGTACTGCGGCTGGCCCACCTTGATGTGCGTCGCCTGGTACCTGCGGGCGAAGATGCTGCAAGCAGCAGATTACGCG-3′ |  |  |
| YK689 | HSV-1 gB-N489Q | 5′-GCCCCCAAACCCCACGCCCCCGCCGCCCGGGGCCAGCGCCCAGGCGTCCGTGGAGCGCATAGGATGACGACGATAAGTAG-3′ | pBS-KanR-ePheS* (This study) | *E. coli* GS1783/pYEbac102Cre (65) |
|  |  | 5′-CGATGGAGGAGGTGGTCTTGATGCGCTCCACGGACGCCTGGGCGCTGGCCCCGGGCGGCGTGCAAGCAGCAGATTACGCG-3′ |  |  |
| YK691 | HSV-1 gB-N674Q | 5′-CCGCGCCGACATCACCACCGTCAGCACCTTCATCGACCTCCAGATCACCATGCTGGAGGAAGGATGACGACGATAAGTAG-3′ | pBS-KanR-ePheS* (This study) | *E. coli* GS1783/pYEbac102Cre (65) |
|  |  | 5′-CCAGGGGGACAAACTCGTGATCCTCCAGCATGGTGATCTGGAGGTCGATGAAGGTGCTGATGCAAGCAGCAGATTACGCG-3′ |  |  |
| YK693 | HSV-1 gB-N888Q | 5′-CAAGGTCACCGACATGGTCATGCGCAAGCGCCGCAACACCCAGTACACCCAAGTTCCCAAAGGATGACGACGATAAGTAG-3′ | pBS-KanR-ePheS* (This study) | *E. coli* GS1783/pYEbac102Cre (65) |
|  |  | 5′-CGTCGGCGTCACCGTCTTTGTTGGGAACTTGGGTGTACTGGGTGTTGCGGCGCTTGCGCATGCAAGCAGCAGATTACGCG-3′ |  |  |
| YK682 | HSV-1 gB-N87Q-repair | 5′-AAAACCGAAAAACCCACCGCCGCCGCGCCCCGCCGGCGACAACGCGACCGTCGCCGCGGGAGGATGACGACGATAAGTAG-3′ | pBS-KanR-ePheS* (This study) | *E*. *coli* GS1783*/* containing the YK681 genome (This study) |
|  |  | 5′-GCTCGCGCAGGGTGGCGTGGCCCGCGGCGACGGTCGCGTTGTCGCCGGCGGGGCGCGGCGTGCAAGCAGCAGATTACGCG-3′ |  |  |
| YK684 | HSV-1 gB-N141Q-repair | 5′-CGAGCAGCCGCGCCGCTGCCCGACCCGGCCCGAGGGTCAGAACTACACGGAGGGCATCGCAGGATGACGACGATAAGTAG-3′ | pBS-KanR-ePheS* (This study) | *E*. *coli* GS1783*/* containing the YK683 genome (This study) |
|  |  | 5′-TGTTCTCCTTGAAGACCACCGCGATGCCCTCCGTGTAGTTCTGACCCTCGGGCCGGGTCGTGCAAGCAGCAGATTACGCG-3′ |  |  |
| YK686 | HSV-1 gB-N398Q-repair | 5′-CCGATTCTCCTCCGACGCCATATCCACCACCTTCACCACCAACCTGACCGAGTACCCGCTAGGATGACGACGATAAGTAG-3′ | pBS-KanR-ePheS* (This study) | *E*. *coli* GS1783*/* containing the YK685 genome (This study) |
|  |  | 5′-CCCCCAGGTCCACGCGCGAGAGCGGGTACTCGGTCAGGTTGGTGGTGAAGGTGGTGGATATGCAAGCAGCAGATTACGCG-3′ |  |  |
| YK688 | HSV-1 gB-N430Q-repair | 5′-CGCCCGCGACGCCATGGACCGCATCTTCGCCCGCAGGTACAACGCGACGCACATCAAGGTAGGATGACGACGATAAGTAG-3′ | pBS-KanR-ePheS* (This study) | *E*. *coli* GS1783*/* containing the YK687 genome (This study) |
|  |  | 5′-GGTAGTACTGCGGCTGGCCCACCTTGATGTGCGTCGCGTTGTACCTGCGGGCGAAGATGCTGCAAGCAGCAGATTACGCG-3′ |  |  |
| YK690 | HSV-1 gB-N489Q-repair | 5′-GCCCCCAAACCCCACGCCCCCGCCGCCCGGGGCCAGCGCCAACGCGTCCGTGGAGCGCATAGGATGACGACGATAAGTAG-3′ | pBS-KanR-ePheS* (This study) | *E*. *coli* GS1783*/* containing the YK689 genome (This study) |
|  |  | 5′-CGATGGAGGAGGTGGTCTTGATGCGCTCCACGGACGCGGTGGCGCTGGCCCCGGGCGGCGTGCAAGCAGCAGATTACGCG-3′ |  |  |
| YK692 | HSV-1 gB-N674Q-repair | 5′-CCGCGCCGACATCACCACCGTCAGCACCTTCATCGACCTCAACATCACCATGCTGGAGGAAGGATGACGACGATAAGTAG-3′ | pBS-KanR-ePheS* (This study) | *E*. *coli* GS1783*/* containing the YK691 genome (This study) |
|  |  | 5′-CCAGGGGGACAAACTCGTGATCCTCCAGCATGGTGATGGTGAGGTCGATGAAGGTGCTGATGCAAGCAGCAGATTACGCG-3′ |  |  |
| YK694 | HSV-1 gB-N888Q-repair | 5′-CAAGGTCACCGACATGGTCATGCGCAAGCGCCGCAACACCAACTACACCCAAGTTCCCAAAGGATGACGACGATAAGTAG-3′ | pBS-KanR-ePheS* (This study) | *E*. *coli* GS1783*/* containing the YK693 genome (This study) |
|  |  | 5′-CGTCGGCGTCACCGTCTTTGTTGGGAACTTGGGTGTAGGTGGTGTTGCGGCGCTTGCGCATGCAAGCAGCAGATTACGCG-3′ |  |  |
| YK695 | HSV-1 ΔgB | 5′-GCCCCCAGGCTACCTGACGGGGGGCACGACGGGCCCCCGTAGTCCCGCCAAGGATGACGACGATAAGTAGGG-3′ | pEP-Kan-S (23) | *E. coli* GS1783/pYEbac102Cre (65) |
|  |  | 5′-TCGCCCATCCCCTCGAAGAACGCGCCCAGGCCCGCGAACATGGCGGCGTTCAACCAATTAACCAATTCTGATTAG-3′ |  |  |
| YK650 | HSV-1 UL51-T190A_KanR/ePheS* | 5'-GCTTGGGGTGACCGAGGCGCCCTCCTTGGGGCACCCCCACGCACCGCCCCCGGAGGTTACAGGATGACGACGATAAGTAGGG-3' | pBS-KanR-ePheS* (This study) | *E. coli* GS1783/pYEbac102Cre (65) |
|  |  | 5'-CGTTTCGGGCGGCAGGCGCCAGCGTAACCTCCGGGGGCGGTGCGTGGGGGTGCCCCAAGGTGCAAGCAGCAGATTACGCG-3' |  |  |
| YK649 | HSV-1 UL51-T190A_KanR | 5'-GCTTGGGGTGACCGAGGCGCCCTCCTTGGGGCACCCCCACGCACCGCCCCCGGAGGTTACAGGATGACGACGATAAGTAGGG-3' | pEP-Kan-S (23) | *E. coli* GS1783/pYEbac102Cre (65) |
|  |  | 5'-CGTTTCGGGCGGCAGGCGCCAGCGTAACCTCCGGGGGCGGTGCGTGGGGGTGCCCCAAGGCAACCAATTAACCAATTCTGATTAG-3' |  |  |
| YK717 | HSV-1 gB-SE | 5′-AGTTCCCAACAAAGACGGTGACGCCGACGAGGACGACCTGGGAGGTTCAGAGAATTTGTA-3′ | pBS-TEV-2xStrep-KanS (This study) | *E. coli* GS1783/pYEbac102Cre (65) |
|  |  | 5′-TTAACACCCGTGGTTTTTATTTACAACAAACCCCCCGTCATTTTTCGAACTGCGGGTGGC-3′ |  |  |
| YK718 | HSV-1 gD-SE | 5′-GGAAGACGACCAGCCGTCCTCGCACCAGCCCTTGTTTTACGGAGGTTCAGAGAATTTGTA-3′ | pBS-TEV-2xStrep-KanS (This study) | *E. coli* GS1783/pYEbac102Cre (65) |
|  |  | 5′-CAGACCTGACCCCCCCGCACCCATTAAGGGGGGGTATCTATTTTTCGAACTGCGGGTGGC-3′ |  |  |
| N/A | HSV-1 ΔUL54_KanR/ePheS* | 5'-ATCCGACACCCCAGCCCCGACGGCAGCCGACAGCCCGGTCGTACAATAAAAACAAAACATAGGATGACGACGATAAGTAGGG-3' | pBS-KanR-ePheS* (This study) | *E. coli* GS1783/pYEbac102Cre (65) |
|  |  | 5'-CGTGGGGCGATTTGTTTGAAATGTTTTGTTTTTATTGTACGACCGGGCTGTCGGCTGCCGTGCAAGCAGCAGATTACGCG-3' |  |  |
| N/A | HSV-1 ΔUL54_KanR | 5'-ATCCGACACCCCAGCCCCGACGGCAGCCGACAGCCCGGTCGTACAATAAAAACAAAACATAGGATGACGACGATAAGTAGGG-3' | pEP-Kan-S (23) | *E. coli* GS1783/pYEbac102Cre (65) |
|  |  | 5'-CGTGGGGCGATTTGTTTGAAATGTTTTGTTTTTATTGTACGACCGGGCTGTCGGCTGCCGCAACCAATTAACCAATTCTGATTAG-3' |  |  |
| N/A | HSV-1 ICP22-flag_KanR/ePheS* | 5'-GCGGGGGGAAGCCACTGTGGTCCTCCGGGACGTTTTCTGGATGGACTACAAAGACGATGACGACAAGATGGCCGAGGATGACGACGATAAGTAGGG-3' | pBS-KanR-ePheS* (This study) | *E. coli* GS1783/pYEbac102Cre (65) |
|  |  | 5'-TTACACAAGGCGCAAAAGCGCCTGGGGAAATGTCGGCCATCTTGTCGTCATCGTCTTTGTAGTCCATCCAGAATGCAAGCAGCAGATTACGCG-3' |  |  |
| N/A | HSV-1 ICP22-flag_KanR | 5'-GCGGGGGGAAGCCACTGTGGTCCTCCGGGACGTTTTCTGGATGGACTACAAAGACGATGACGACAAGATGGCCGAGGATGACGACGATAAGTAGGG-3' | pEP-Kan-S (23) | *E. coli* GS1783/pYEbac102Cre (65) |
|  |  | 5'-TTACACAAGGCGCAAAAGCGCCTGGGGAAATGTCGGCCATCTTGTCGTCATCGTCTTTGTAGTCCATCCAGAACAACCAATTAACCAATTCTGATTAG-3' |  |  |
